# Inhibition of the PI3K signaling pathway in cancer cells by *Agrimonia eupatoria* L. ethanolic extract: identification of tricoumaroyl spermidine as a potential PI3K inhibitor

**DOI:** 10.1186/s12906-025-05231-z

**Published:** 2026-01-06

**Authors:** Mikayel Ginovyan, Smbat Gevorgyan, Hayarpi Javrushyan, Barbara Kusznierewicz, Izabela Koss-Mikołajczyk, Naira Sahakyan, Agnieszka Bartoszek, Nikolay Avtandilyan

**Affiliations:** 1 Research Institute of Biology, Yerevan State University, 1 Alex Manoogian, Yerevan, 0025 Armenia; 2 Denovo Sciences Inc, Yerevan, Armenia; 3 Laboratory of Antiviral Drug Discovery, Institute of Molecular Biology of NAS RA, 7 Ezras Hasratyan St, Yerevan, Armenia; 4 Department of Biochemistry, Biology Faculty, Microbiology and Biotechnology, Yerevan State University, Alex Manoogian 1, 0025 Yerevan, Armenia; 5 Department of Food Chemistry, Faculty of Chemistry, Technology and Biotechnology, Gdańsk University of Technology, Narutowicza 11/12, 80-233 Gdańsk, Poland

**Keywords:** PI3K signaling pathway, Cancer therapy, Phytochemical profiling, Molecular docking, Antioxidant activity, Tricoumaroyl spermidine

## Abstract

**Background:**

Cancer remains one of the most significant global health challenges, requiring continuous efforts to identify novel anticancer agents. *Agrimonia eupatoria* L. (AE), is a perennial herb with diverse therapeutic properties, showing promise in preclinical studies for its anticancer potential. The aim of this study was to investigate the inhibitory effect of the AE extract on cancer cells in vitro and assess its impact on the phosphoinositide 3-kinase (PI3K) signaling pathway, a key regulator of cancer-related processes and one of its potential targets.

**Methods:**

Metabolomic profiling of the AE ethanol extract composition was done using an advanced LC-Q-Orbitrap HRMS technique. The MTT assay was used to assess the cytotoxicity of the AE extract against four human cancer cell lines (MCF-7, HT-29, A549, HeLa). PI3K signaling pathway was elucidated with an In-Cell ELISA assay, WB, ICC/IF, and molecular docking identified potential PI3K inhibitors.

**Results:**

MTT results showed significant cytotoxicity of the AE extract across all tested cell lines. A cellular antioxidant activity assay revealed a pro-oxidant effect in cancer cells, a process linked to PI3K/Akt regulation. The AE extract reduced both total and phosphorylated PI3K, and indicating inhibition of the PI3K/Akt/mTOR/COX-2/MMP-2/HIF1a axis. Molecular docking identified tricoumaroyl spermidine as a potential PI3K inhibitor with high binding affinity.

**Conclusion:**

These findings support the potential of AE as a source of novel anticancer agents. However, this study has limitations, being confined to in vitro and in silico models; the extract’s overall biological activity is likely due to the synergistic effects of multiple constituents. Future in vivo validation and pharmacokinetic studies are necessary. Despite these challenges, tricoumaroyl spermidine was identified as a promising lead compound for further development as a PI3K inhibitor.

**Graphical Abstract:**

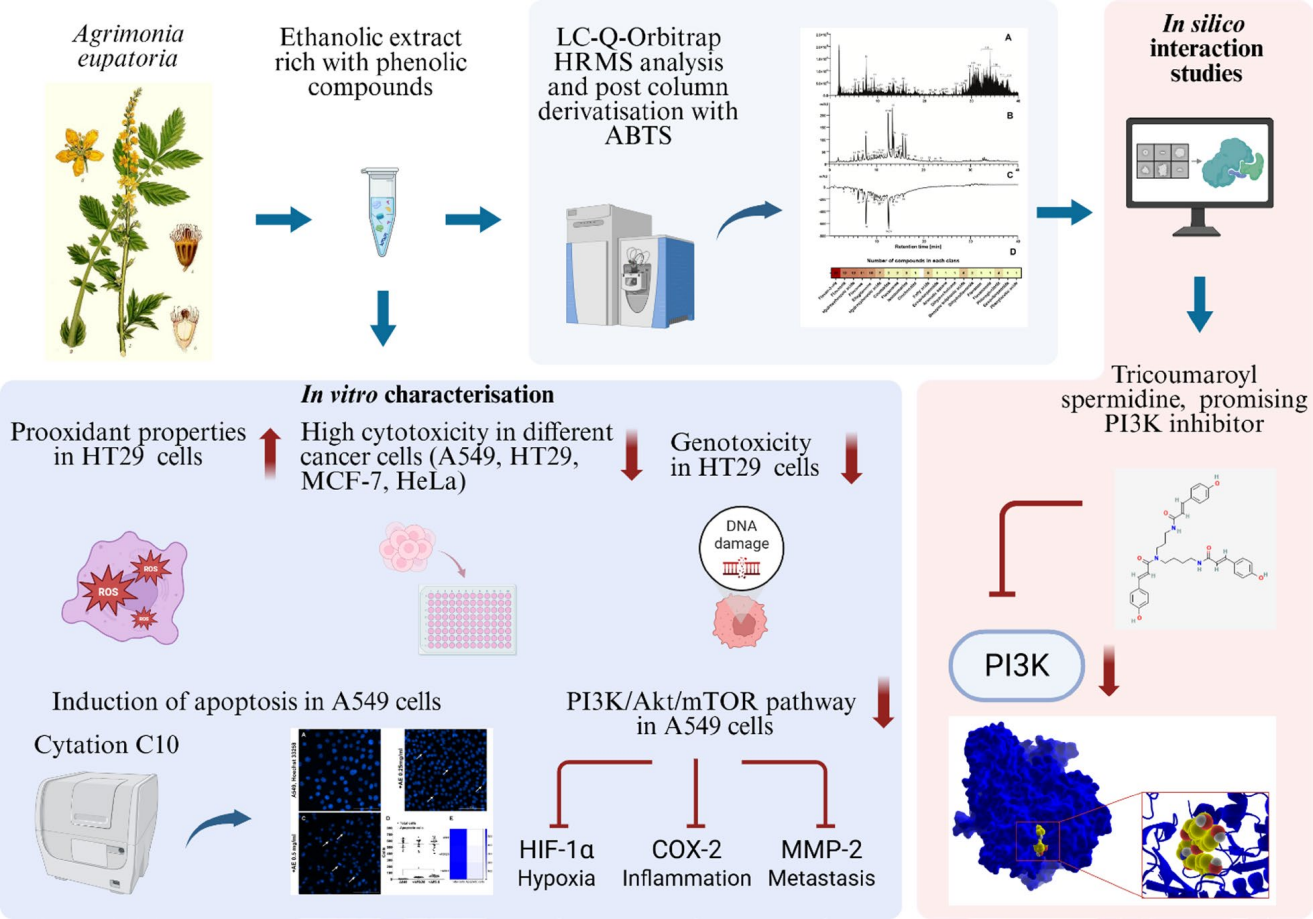

**Supplementary Information:**

The online version contains supplementary material available at 10.1186/s12906-025-05231-z.

## Introduction

Cancer remains one of the most significant health challenges, with its incidence continuously rising worldwide. In 2022 alone, approximately 20 million new cancer cases and 9.7 million cancer-related deaths were recorded globally [1]. Despite significant improvements in treatment, the demand for novel and effective anticancer agents remains high [1, 2]. One promising approach lies in ethnopharmacology, which draws on the traditional use of plant-derived substances to identify new therapeutic leads. The shift away from the ‘one-disease, one-target’ approach towards using plant compounds that target multiple physiological processes represents a new paradigm called polypharmacology [3].

*Agrimonia eupatoria* L. (AE), or common agrimony, is a perennial herb with a rich history in European and Asian folk medicine. It has been traditionally used for a wide range of ailments, including inflammatory conditions, infections, and digestive disorders, including for potential anticancer properties. Several studies reported its cytotoxic activity against different cancer cell lines in vitro [4–7]. Some authors have also reported the antitumor effect of agrimonin – one of the main components of *A. eupatoria* extract [8–10]. Common agrimony has been traditionally used in folk medicine for its biological properties, including anti-inflammatory, immunomodulatory, antioxidant, analgesic, antidiabetic, wound healing, diuretic, hepatoprotective, antiviral, anticancer, and antimicrobial effects [5, 6, 11]. It has been used for the treatment of migraines, sore throats, bronchitis, eczema, bleeding disorders, varicose ulcers, conjunctivitis, cystitis, urinary infections, diarrhea, wounds, sores, and conditions involving excess phlegm or mucus, hepatitis, nephritis, stomatitis, and yellow fever [5, 6, 12]. The Committee on Herbal Medicinal Products (HMPC) has recognized agrimony for its traditional use in several applications based on its long-standing utilization and there is a separate document of the European Medicines Agency (EMA) describing this [13]. According to this document, the HMPC based on traditional uses of this plant concluded that it can be used by adults and children over the age of 12 years for the relief of mild diarrhea, or used as gargle for the treatment of oral cavity and for relief of skin minor inflammations and wounds (https://www.ema.europa.eu/en/medicines/herbal/agrimoniae-herba). There are several pharmaceutical forms of *A. eupatoria* medical preparations such as comminuted herbal substance as herbal tea for oral use, comminuted herbal substance for infusion preparation or decoction preparation for oromucosal use, cutaneous use or use as a bath additive and herbal preparations in liquid dosage forms for oral or oromucosal use (Assessment report on *A. eupatoria* L., herba EMA/HMPC/680595/2013). Currently, investigations are ongoing to explore different bioactive properties of AE extracts, with the aim of proposing more uses for the herb and revising the relevant monograph.

Investigations have revealed the presence of diverse bioactive compounds within *A. eupatoria* extracts, including phenolic acids, flavonoids, tannins, polysaccharides, volatile oils, terpenoids, etc [6, 11]. Some literature data state that the main polyphenolic compound in aerial parts of common agrimony is agrimoniin [14]. It is also abundant with flavonoid glycosides, such as apigenin, luteolin, quercetin, etc. These constituents exhibit a spectrum of pharmacological activities, including cytotoxic, apoptotic, and antiangiogenic effects, which can be promising in combating cancer progression. These findings suggest significant potential for developing new medicinal applications based on AE bioactive constituents [5].

Our interest was specifically driven by previous findings on AE extracts sourced from the high altitudes of Armenia. Our earlier studies demonstrated that the Armenian ecotype of AE possesses significant antiradical and antiviral properties [12, 15]. Furthermore, we found it has compounds with potent antibacterial activity that can enhance the efficacy of antibiotics against resistant bacterial strains [16]. While these results and studies from other research groups confirm AE’s cytotoxic potential in vitro, the precise molecular mechanisms behind its anticancer activity have not been sufficiently explored. There is a clear need to identify the specific bioactive components responsible for these effects and to understand how they modulate key cellular pathways in cancer. Furthermore, comprehensive phytochemical profiling is imperative to identify and characterize the bioactive components responsible for the observed anticancer effects. Considering that, we were aimed to further explore the mechanisms of AE action at the molecular level and define the compounds responsible for this.

One of the most critical signaling axes in cancer is the phosphoinositide 3-kinase (PI3K) pathway, which governs processes like cell proliferation, survival, metastasis, and drug resistance. Aberrations in this pathway are common drivers of tumorigenesis in many cancers, making it a strategic target for therapy [17, 18]. Aberrations in the PI3K pathway, such as mutations or amplifications in PI3K subunits, are commonly observed in various cancers, contributing to tumorigenesis and resistance to conventional therapies [17–19]. For instance, the occurrence of PIK3CA mutations is an initial event in the development of both breast and colon cancer [17, 19]. Decreased expression of PIK3CG has been associated with the promotion of colon cancer growth and development [18]. Therefore, targeting PI3K signaling represents a strategic approach to combat cancer [17, 18]. The PI3K/Akt pathway is also closely regulated by cellular redox metabolism and oxidative stress. This link provides a compelling rationale for investigating whether the antiradical properties we previously observed in AE extract could be related to a modulatory effect on PI3K signaling [20, 21].

Therefore, this study aimed to further explore the anticancer potential of the ethanolic AE extract, focusing specifically on its ability to modulate the PI3K/Akt/mTOR signaling pathway and its downstream targets. By combining advanced phytochemical profiling with in vitro mechanistic studies and in silico molecular docking, we sought to identify the key bioactive compounds responsible for its action and elucidate their molecular interactions.

## Materials and methods

### Chemicals and reagents

Folin–Ciocalteu (FC) reagent, ethanol, gallic acid, catechin 3-(4,5-dimethylthiazol-2-yl)−2,5-diphenyltetrazolium bromide (MTT), McCoy’s 5 A Medium, Minimal Essential Medium Eagle (MEM), Dulbecco’s Modified Essential Medium (DMEM), phosphate-buffered saline (PBS), ethanol, 2-amino-2-(hydroxymethyl)−1,3-propanediol (Trizma-Base), Sybr Green I nucleic acid gel stain, Triton X100, low and normal melting point agarose (LMP and NMP agarose), 2,2-azinobis-(ethyl-2,3-dihydrobenzothiazoline-6-sulfonic acid) diammonium salt (ABTS), and other chemicals were purchased from Sigma-Aldrich GmbH (Taufkirchen, Germany). The OxiSelect™ Cellular Antioxidant Assay Kit was purchased from Cell Biolabs, Inc. Universal Mycoplasma Detection Kit was acquired from ATCC (USA). The antibodies PI3K/AKT signalling pathway panel (ab283852), HIF1a (ab179483), MMP-2 (ab92536), COX-2 (ab179800), Goat anti-rabbit IgG H&L (Alexa Fluor488, ab150077), anti-beta actin (ab8227), anti-Caspase-3 (ab32351), and anti-rabbit IgG H&L (HRP, ab6721) for WB and ICC/IF were purchased from Abcam.

### Plant material

The aerial parts of *Agrimonia eupatoria* L. (AE) (Rosaceae family), a perennial plant widely distributed throughout Armenia, were harvested from the Tavush region, near the village Navur in Armenia, at an elevation of 1400–1550 m above mean sea level, during the plant’s flowering period at June 2023. The sampling was done from a private grassland. The landowner provided permission for the sampling. The identification of the plant material was carried out by Dr. Narine Zakaryan at the Department of Botany and Mycology, Yerevan State University (YSU), Armenia. Voucher specimens were deposited in the Herbarium of Yerevan State University (Yerevan, Armenia) under the serial number: Agrimonia eupatoria L. (ERCB 13207).

### Plant extract preparation

The AE samples used in the experiments were prepared through maceration using 96% ethanol in a 1:10 (w/v) ratio of plant dry material to solvent volume, following the method described previously [22]. Maceration was performed at 2–8 °C for 24, and the process was repeated three times to ensure exhaustive extraction. For the CAA (Cellular Antioxidant Activity), MTT (3-(4,5-dimethylthiazol-2-yl)−2,5-diphenyltetrazolium bromide), and comet assays, a crude ethanol extract of 50 mg DW (dried weight)/mL was prepared. The extraction yield was determined by evaporating 500 µL of the extract at room temperature under ambient conditions and then weighing the dry residue. This process was repeated in three independent experiments, resulting in an average yield of 15.4 ± 0.28%.

### Cell culture

MCF-7 (ATCC HTB‐22) (human breast cancer), HT-29 (ATCC HTB‐38) (Human colon adenocarcinoma), A549 (ATCC CCL-185) (human lung adenocarcinoma) and HeLa (ATCC CCL-2) (human cervical carcinoma) CCD 841 CoN (ATCC CRL-1790) (non-cancerous human colon fibroblast cells) cell cultures were obtained from ATCC. MCF‐7 and CCD 841 CoN cells were maintained in Minimal Essential Medium Eagle (EMEM), HT-29 in McCoy’s medium, whereas A549 and HeLa cells in Dulbecco’s Modified Essential Medium (DMEM). All used mediums were supplemented with 10% with fetal bovine serum (100 mL/L), sodium pyruvate (200 mg/L), L‐glutamine (2 mmol/L), and antibiotics (100 µg/mL streptomycin, 100 U/mL penicillin, and 0.25 µg/mL of Amphotericin B). The cells were grown at 37 °C and a humidified atmosphere with 5% CO2 in a Biosmart (Biosan, Latvia) incubator or/and in a Smart Cell Incubator (Heal Force), following the previously described protocol [23, 24]. Cultured cells were regularly examined for the presence of mycoplasma contamination using the Universal Mycoplasma Detection Kit from ATCC (USA). All cell line experiments were conducted in accordance with institutional guidelines for in vitro research. The passage numbers of the cell lines used were between 5 and 15.

### Antioxidant activity by chemical assays and quantification of total phenolic and flavonoid contents

The antioxidant activity of the AE extract was assessed using standard spectrophotometric methods with ABTS (2,2′-azino-bis-(3-ethylbenzothiazoline-6-sulfonic acid) and DPPH (1-diphenyl-2-picrylhydrazyl) radicals, following previously described procedures [25, 26]. For the measurements, stock solutions of both radicals were prepared as follows: the 0.1mM DPPH solution was diluted with methanol to achieve an absorbance of 0.9 ± 0.02 at 515 nm, and the 0.05 mM ABTS solution was diluted with ethanol to reach an absorbance of 0.7 ± 0.02 at 734 nm. The diluted reagents were then mixed with various dilutions of the test samples, and absorbance was measured at 515 nm for DPPH and 734 nm for ABTS after a 10-minute incubation at room temperature. All measurements were performed using a TECAN Infinite M200 Spectrophotometer (Tecan Group Ltd., Männedorf, Switzerland). The stoichiometry values indicating the number of oxidant molecules reduced by one molecule of antioxidant after 10 min of reaction for the ABTS or DPPH assays (n10) were measured at room temperature. The regression coefficient was calculated as the slope of the line representing the relationship between the amount of DPPH/ABTS scavenged (µg) by a radical scavenger and the quantity of the tested antioxidant extract (µg) in the mixture after 10 min of reaction (n10).

The evaluation of the total phenolic content in plant extracts was assessed using the Folin–Ciocalteu (FC) reagent, with the quantification based on a calibration curve prepared for gallic acid (GA), concentrations ranging from 0 to 250 µg/mL. The phenolic content was calculated as milligrams of gallic acid equivalents per gram of the dry weight of the plant sample (mg GAE/g). For the total flavonoid content estimations, the AlCl_3_ colorimetric assay was used. The flavonoid levels were quantified based on a calibration curve created for quercetin, concentrations spanning from 0 to 250 µg/mL. The results were presented as milligrams of quercetin equivalents per gram of the plant sample’s dry weight (mg QE/g).

### Cellular antioxidant activity assay

The cellular antioxidant activity (CAA) of the AE aerial part extract was evaluated in HT-29 cells employing the OxiSelect Cellular Antioxidant Activity Assay Kit (green fluorescence), following previously described methods [25]. Briefly, cells were cultured until 90% confluence was achieved. They were then treated with 50 µL of plant extracts for 1 hour. In addition, 50 µL of the fluorescent probe 2’,7’-dichlorodihydrofluorescein diacetate (H2DCFDA) was added. Following the introduction of a radical initiator, which is AAPH (2,2’-Azobis(2-amidinopropane) dihydrochloride), the resultant fluorescence intensity, which reflects the levels of reactive oxygen species (ROS), was measured. The fluorescence emission was detected at 538 nm after excitation at 485 nm, with measurements taken every 5 min over 1 h, using a TECAN Infinite M200 plate reader (Tecan Group Ltd., Männedorf, Switzerland).

### MTT cytotoxicity assay

The MTT assay was conducted to evaluate the inhibitory effect of AE extract on the growth of MCF-7, HT-29, A549, HeLa and CCD 841 CoN cells after different exposure times to various concentrations of extract, as previously described [22]. 1% ethanol was used as control. Three independent experiments were conducted with three or four technical replicates in each. Cytotoxicity was quantified as the percentage of growth inhibition in cells treated with the test plant extract compared to control cells treated solely with the appropriate volume of solvent (1% ethanol in the final mixture), the growth of which was considered 100%. The final concentration of the vehicle used was determined to be non-toxic to the cells. The IC_50_ values for the extract, across different exposure times, were determined using a nonlinear regression curve fit with a variable slope.

### Comet assay

The genotoxicity of the AE extract was evaluated in the HT-29 cell line using the single-cell gel electrophoresis, or Comet assay, following previously established procedures [25–27]. Approximately 10^5^ cells per well were seeded into 24-well tissue culture plates and were subsequently treated with various concentrations of the plant extract for a 24-hour period. Following the incubation, cells were harvested and embedded in a layer of low-melting-point agarose on microscope slides. The cells were then lysed using a high-salt detergent solution to remove cell membranes, cytoplasm, and proteins, leaving the nuclear DNA. To detect DNA strand breaks, the slides were immersed in an alkaline buffer to unwind the DNA. Electrophoresis was then performed under these alkaline conditions, causing the negatively charged, fragmented DNA to migrate from the nucleoid (the comet head) towards the anode, forming a tail. The amount of DNA that migrates into the tail is directly proportional to the extent of DNA damage. After electrophoresis, the slides were neutralized, and the DNA was stained with a fluorescent dye. The resulting comets were visualized using fluorescence microscopy. The genotoxic potential of the extract was quantified by analyzing the images to determine the average percentage of DNA present in the comet tail. This entire assessment was conducted in two independent experiments, with each experiment comprising three technical replicates to ensure the reliability of the findings.

### LC-Q-Orbitrap HRMS analysis and post-column derivatization with ABTS

The phytochemical constituents of the AE ethanol extract were characterized using a Dionex Ultimate 3000 UHPLC system (ThermoScientificTM, Dionex, San Jose, CA, USA) equipped with a Luna Omega Polar C18, 100 Å (150 × 2.1 mm, 1.6 μm, Phenomenex) column, following the methodology outlined by Kusznierewicz et al. [28]. The elution was performed with water (A) and acetonitrile (B) as the mobile phase, each containing 0.1% v/v formic acid, at a flow rate of 0.3 mL/min and an injection volume of 2 µL for all samples. The gradient elution schedule was set as follows: starting at 15% B, increasing to 40% B over 15 min, then to 100% B at 16 min, maintaining 100% B until 24 min, followed by a return to the initial conditions for 7 min to re-equilibrate. This chromatographic setup was linked to a Q Exactive TM Focus quadrupole-Orbitrap mass spectrometer (Thermo Fisher Scientific, Bremen, Germany) equipped with a heated electrospray ionization source (HESI II). Detection was carried out using the Q-Exactive mass spectrometer under the following conditions for negative ion mode: sheath gas flow rate at 35 arb, auxiliary gas flow rate at 15 arb, sweep gas flow rate at 3 arb, spray voltage at 2.5 kV, capillary temperature at 350 °C, S-lens RF level at 50, and heater temperature at 300 °C. Full-scan analysis parameters included a resolution of 70,000, AGC target of 1e6, automatic max IT, and a scan range of 120–1200 m/z. For data-dependent MS2 analysis, the settings were: resolution of 17,500, isolation window of 3.0 m/z, normalized collision energy of 30, AGC target of 1e6, and automatic max IT. High-resolution mass spectrometry raw data were processed using Compound Discoverer software (v. 2.1, Thermo, Waltham, USA).

The identification of major metabolites from the high-resolution mass spectrometry raw data was performed using Compound Discoverer (v. 2.1, Thermo, Waltham, USA). Metabolites were identified based on their precise mass (with a tolerance of 5 ppm), retention time behavior (tolerance of 0.1 min), and fragmentation pattern. The workflow involved database searches using ChemSpider, mzCloud, mzVault (February 2017), and locally curated mass lists compiled from literature on the studied plants. All annotations produced by Compound Discoverer were rigorously evaluated by comparing the MS/MS spectra against reference databases (such as HMDB) and by assessing the likelihood of each metabolite’s presence in the specific plant matrix based on established phytochemical knowledge.

The antioxidant profiles of the AE extract were determined using an HPLC-DAD system (Agilent Technologies, Wilmington, DE, USA), which was integrated with a Pinnacle PCX Derivatization Instrument (Pickering Laboratories Inc., Mountain View, CA, USA) and a UV–Vis detector (Agilent Technologies, Wilmington, DE, USA). The chromatographic separation conditions mirrored those used in the LC-HRMS analysis. Initial chromatograms were captured at 270 nm using a DAD detector before the eluate was routed to the post-column derivatization equipment. The derivatization process employed the ABTS reagent, adapted from methods reported in the literature with minor modifications [29]. A methanolic ABTS solution (1 mM) was merged with the eluate at a flow rate of 0.1 mL/min, then sent through a reaction loop (1 mL, 130 °C). The resulting antioxidant profiles were detected with a UV-Vis detector at 734 nm.

### Morphological analysis of apoptosis by Hoechst 33258 staining

Hoechst 33258 staining enables the determination of apoptotic cells through morphological analysis using a fluorescence microscope (x400 magnification, Cytation C10, Agilent, USA) [30]. Briefly, A549 cells (2 × 10^5^ cells/mL) were incubated with vehicle (1% ethanol in PBS), and test samples: AE (0.25 mg/mL), AE (0.5 mg/mL) for 24 h. Cells were fixed with 4% paraformaldehyde in PBS for 10 min, washed twice with PBS for 5 min, and stained with Hoechst 33,258 (10 𝜇g/mL) for 10 min in the dark. Cells with typical morphological nuclei changes, such as chromatin condensation, rough edges, nuclear fragmentation, and apoptotic bodies were counted as apoptotic. Controls and treatment variants were examined in triplicate. For each variant, 8 fields were examined, and the total cell count and apoptotic cells were calculated using Gen5 software. The statistical analysis of was performed by Two-way ANOVA using Sidak’s multiple comparisons test.

### Phospho-PI 3 kinase p85 + total in-cell ELISA assay

A549 cells were seeded in the 96-well plates (1.5 × 10^4^ cells per well). After 24 h incubation, the cell medium (180 𝜇L) was replaced with the fresh one and the cells were treated with 20 𝜇L negative control or test extracts with the following final concentrations: PBS containing 1% ethanol (Control, A549), *A. eupatoria* extract (0.25 mg DW/mL). The calculations during the seeding of the cells were performed to ensure that the cells reached approximately 80% confluence at the time of fixation. After 24 h of exposure to the test extracts or controls, the medium was removed, and the cells were fixed using 100 µL of 4% formaldehyde in PBS. Crystal violet staining was then applied to the cells to normalize the readings at 450 nm for Phospho-PI 3 kinase p85 and Total PI3K. The measured OD450 readings were corrected for cell number by dividing the OD450 reading for a given well by the OD595 reading for that well. This relative cell number was then used to normalize each reading. Total and phospho-PI 3 kinase p85 were each assayed in triplicate using the phospho- and total PI 3 Kinase p85 antibodies included in the PI 3 Kinase Kit. Levels of Phospho-PI 3 kinase p85 and Total PI3K were measured using an In-Cell ELISA kit (ab207484, Abcam), according to the manufacturer’s instructions.

### Western blots

A549 cells (5 × 10^5 cells per well) were cultured in 6-well plates and incubated for 24 h. Then, the cell medium (900 µL) was refreshed, and the cells were treated with 100 µL of PBS + 1% ethanol solution, 1% ethanol (control), or test compounds at the following final concentrations in these groups: (1) Control cells (ethanol), (2) Control cells + 200nM Insulin, (3) Cells + 200nM Insulin + AE (0.5 mg/mL). After 24 h, the cells were harvested and suspended in RIPA buffer supplemented with protease and phosphatase inhibitor cocktail (Abcam). Total protein concentrations in the fraction were determined using the bicinchoninic acid (BCA) (Bio-Rad Laboratories). Total proteins (20 µg) were separated on 4–20% precast linear gradient gels (Bio-Rad Laboratories), transferred to nitrocellulose membranes, and blocked with 5% (w/v) nonfat milk in TBST for 1 h. Membranes were incubated overnight at 4 °C with the primary antibody (PI3K 1:1000, Akt 1:5000, mTOR 1:2500, β-actin 1:2500) diluted in 1% nonfat milk (w/v) in TBST and detected using an appropriate peroxidase-conjugated secondary antibody (anti-rabbit IgG HandL (HRP) 1:10000) [31]. Products were visualized by ECL chemiluminescence (Millipore). Band intensities were measured using the Chemidoc (Bio-Rad Laboratories). ImageJ software was used to measure the intensities of the protein bands.

### Immunocytochemistry (ICC)

A549 cells (15000) were plated in a 96-well black cell culture microplate and incubated for 24 h. For ICC analysis, the groups were: (1) Control cells (ethanol), (2) Cells + 200nM Insulin, (3) Cells + 200nM Insulin + AE (0.5 mg/ml). Insulin was applied for 8 h, after which the medium was replaced with fresh medium, and the tested plant extract was added. The cells were then incubated for 16 h, followed by fixation. After the indicated treatment, cells were fixed with 4% Paraformaldehyde (PFA), permeabilized with 1% Triton X-100, blocked with 1% BSA, and incubated with primary antibody (PI3K 1:500, Akt 1:250, mTOR 1:250, Caspase-3 1:500, HIF1a 1:250, MMP-2 1:250, COX-2 1:250) at 4 °C overnight. Then, cells were incubated with green fluorescence-labeled secondary antibody (anti-rabbit IgG H&L (Alexa Fluor488) 1:500) and nuclei stained blue with Hoechst (Sigma). Images were visualized on a Cytation C10 confocal microscope using a 20× objective at room temperature and acquired by Gen5 software. Gen5 software was used to analyze average fluorescence intensity.

### Computational preparation of protein crystallographic structure

The crystallographic structure of phosphoinositide 3-kinase (PI3K) was obtained from the Protein Data Bank (PDB) (ID: 6C1S) [32, 33]. The structure of was PI3K analyzed and visualized using the PyMOL Molecular Graphics System (Schrödinger, LLC). Initial preprocessing included the removal of extraneous components such as water molecules, ions, and non-protein entities. Additionally, any bound ligands were removed to isolate the protein coordinates. The refined protein structure was then prepared for subsequent molecular docking studies, while the extracted ligand was reserved for further redocking.

### Molecular docking with AutoDock Vina

AutoDock Vina, a widely-used molecular docking software, employs the “Iterated Local Search global optimizer” to generate docking poses, similar to the optimizers used in ICM and the Broyden-Fletcher-Goldfarb-Shanno (BFGS) quasi-Newton method for local refinement. This program integrates an empirical and knowledge-based scoring function to evaluate binding affinities [34, 35]. For the docking simulations, the “exhaustiveness” parameter was set to 16, and default settings recommended by the developers were used to ensure result accuracy. Subsequently, AutoDock Vina was utilized to evaluate the binding affinities of key compounds derived from AE against the target protein, facilitating the ranking of these compounds based on their predicted binding strengths.

### Calculation of ADME properties

The absorption, distribution, metabolism, and excretion (ADME) properties of the selected ligands were calculated using the SwissADME web server (http://www.swissadme.ch/). This tool provides a comprehensive analysis of ADME characteristics based on molecular descriptors and predictive models.

### 2D interaction analysis

The software Discovery Studio by Biovia was employed to calculate the 2D interactions within the binding pocket of PI3K and tricoumaroyl spermidine. This analysis enabled detailed visualization of the molecular interactions, highlighting key residues involved in ligand binding.

### Statistical analysis

The data are presented as means ± SD. The assessment of antioxidant efficacy in both chemical (ABTS, DPPH) and cellular (CAA) assays was carried out using an unpaired Student’s t-test with a significance threshold of *p* ≤ 0.05. The significant difference for other tests was determined through one-way ANOVA with either Dunnett’s or Tukey’s post-test. Statistical analysis was conducted using GraphPad Prism 8 software (GraphPad Software, Inc., San Diego, CA, USA), considering a p-value < 0.05 as indicative of statistical significance.

## Results

### The antioxidant potential of AE extract

The total content of flavonoids and phenolics in *Agrimonia eupatoria* L. extracts were quantified using standard colorimetric methods. According to obtained data, the flavonoid and phenolic contents were 26.23 ± 0.85 mg (QE)/g and 358.9 ± 0.62 mg (GAE)/g, respectively.

**Fig. 1:**
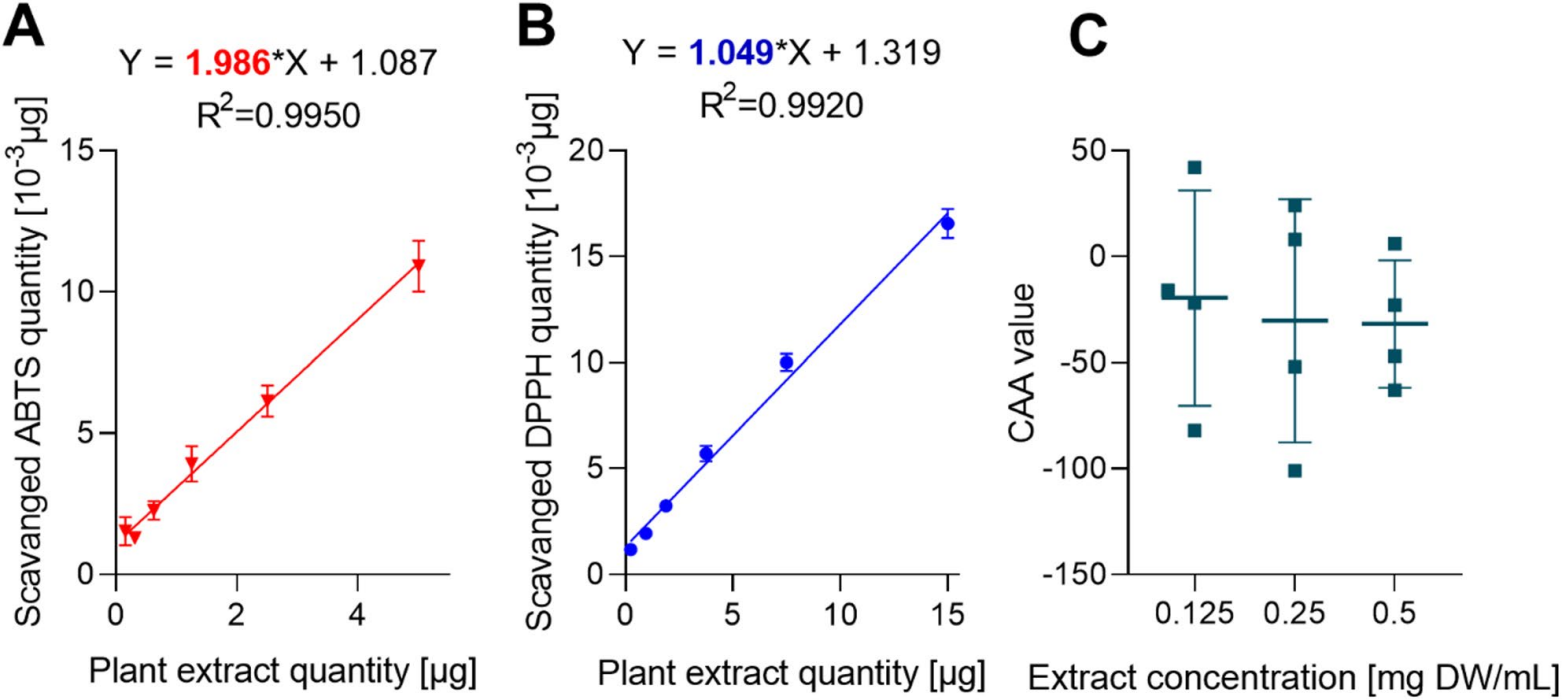
The total antioxidant activity of the *A. eupatoria* aerial part extract was determined by spectrophotometric tests: **A** – ABTS and **B** – DPPH and **C** – in vitro cellular antioxidant activity (CAA) test which was determined in HT-29 cells exposed to the AE extract for 1 h. CAA result is expressed as fluorescence intensity, which is proportional to intracellular oxidation. The results represent means ± SD from at least three independent determinations, *p* < 0.05

The antioxidant potential of AE extract was evaluated using standard spectrophotometric methods - DPPH and ABTS (Fig. 1A and B). The regression coefficient, reflecting stoichiometric values, was calculated from the slope derived from the relationship between the extract’s concentration and the number of radicals scavenged in these chemical-based tests. The calculated stoichiometric values were 1.049 for DPPH and 1.986 for ABTS, indicating that 1 µg of AE crude extract neutralizes 1.049 ng of DPPH or 1.986 ng of ABTS radicals (Fig. 1A, B).

The AE extract displayed average antioxidant properties in chemical tests and significant phenolic and flavonoid content, but the cellular antioxidant activity determined in cell culture using CAA assay revealed contrasting results. The results of assay demonstrated that the AE extract exhibits mainly prooxidant properties in HT-29 cells, depending on the physiological state, redox homeostatic and metabolic status of the cell (Fig. 1C). The CAA assay is designed to mirror the biological conditions with a pH of 7.4 and a temperature of 37 °C, and it considers the bioavailability, distribution, and cellular metabolism of antioxidants, making it more biologically relevant than chemical assays. Thus, AE ethanol extract exhibited a pro-oxidant effect in the cellular model despite its chemical antioxidant profile.

### Cytotoxic properties of *A. eupatoria* extract tested by MTT assay

**Fig. 2:**
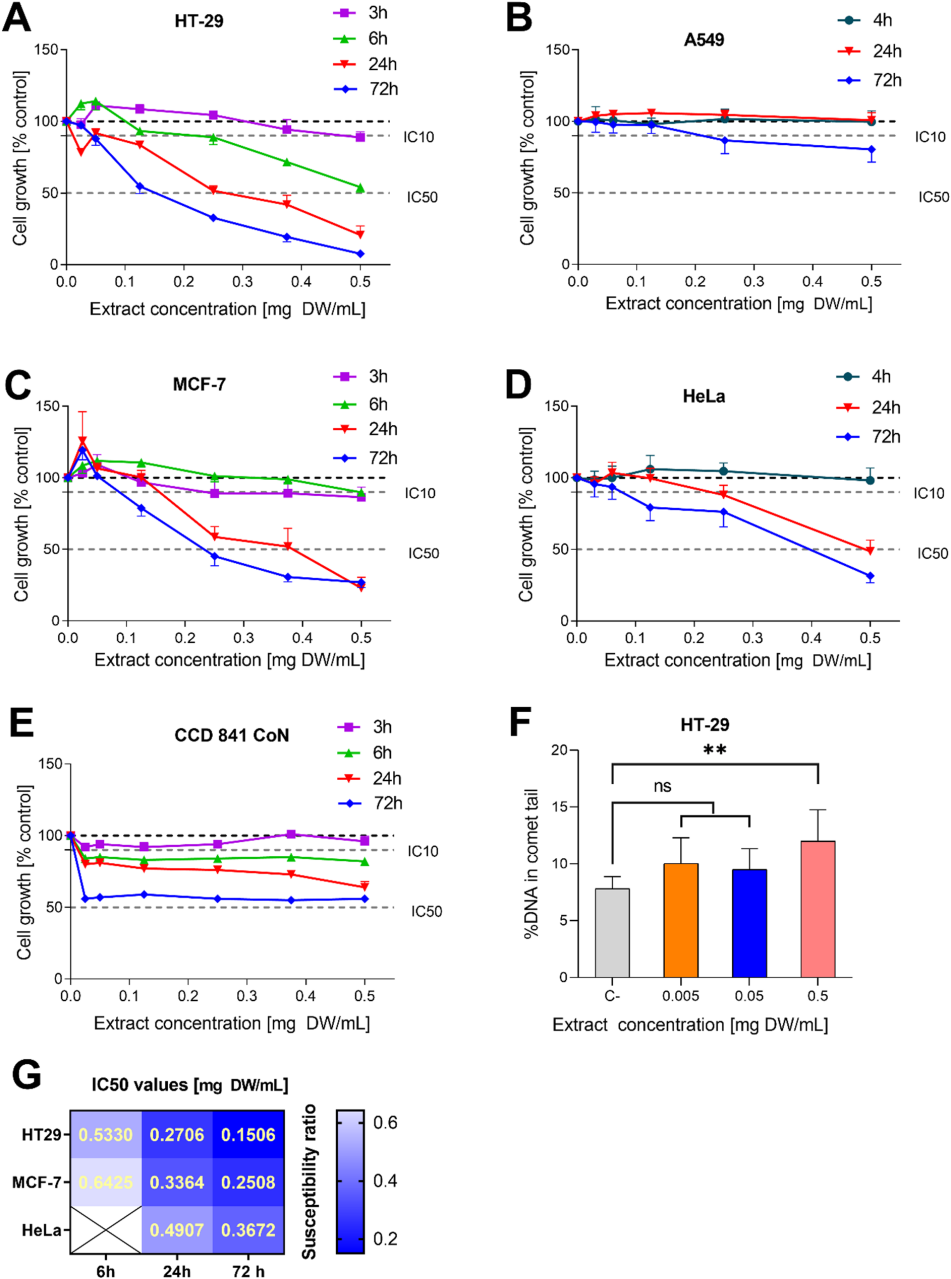
Growth inhibition of HT-29 (panel **A**), A549 (panel **B**), MCF-7 (panel **C**), HeLa (panel **D** and CCD 841 CON (panel **E**) cells treated with AE extracts for different exposure times assessed using MTT assay. The grey broken lines in the graphs mark the level of IC10 and IC_50_ values corresponding to 90% and 50% cell growth, respectively, compared to control (100%). The heatmap of IC_50_ values on AE extract on different cell lines (panel **G**). The genotoxic effects of AE on HT-29 cells expressed as % DNA in the comet tail (panel **F**). Results represent means ± SD from three independent experiments carried out in triplicates. The p-value for 0.5 mg DW/mL concentration was 0.0060

The growth inhibition potential of the AE extract was evaluated against a panel of human cancer cell lines (MCF-7, HT-29, A549, and HeLa), revealing statistically significant concentration- and time-dependent cytotoxicity across all lines (Fig. 2A-D), although shorter exposure times (≤ 4 h) were found to be ineffective. A heatmap analysis of the IC₅₀ values revealed that HT-29 cells were the most susceptible to the AE extract, followed by MCF-7, HeLa, and A549 cells (Fig. 2E). Where IC₅₀ values could not be determined from the dose-response curves, they have been omitted.

Importantly, to assess the selectivity of the extract, its effects were also studied on the non-cancerous human colon fibroblast cell line, CCD841. The results demonstrated that the AE extract was significantly more cytotoxic to the tested almost all cancer cells than to the normal CCD 841 CON fibroblasts, indicating a favorable therapeutic window (Fig. 2E). This selectivity was further emphasized by the time-dependent nature of the effect on normal cells, significant cytotoxicity was only observed after 72 h of incubation, with no notable impact on growth at earlier time points (3, 6, and 24 h). Taken together, these findings highlight the selective growth-inhibitory properties of the AE extract, making it a strong candidate for further development as an anticancer agent.

### Assessment of the genotoxic properties of AE extract by the comet assay

The genotoxic effect of *A. eupatoria* extract was evaluated using the comet assay in HT-29 cells to determine the extent of DNA damage. The increased percentage of DNA in the comet tail compared to the control group was used as a marker for genotoxicity. Our results revealed that at the highest tested concentration of the AE extract (0.5 mg DW/mL) exhibited modest but statistically significant genotoxic properties, with an average of about 12% DNA in the comet tail, compared to the control group, which had an average of about 7.9% (*p* < 0.006) (Fig. 2F). This indicates a marked increase in DNA damage in cells treated with the plant extract. The lower tested concentrations of extract did not exhibit statistically significant genotoxic effects compared to the negative control (Fig. 2F).

### Identification of phenolic compounds within the AE extract and antioxidant profiling by HPLC coupled with post-column derivatization

**Fig. 3:**
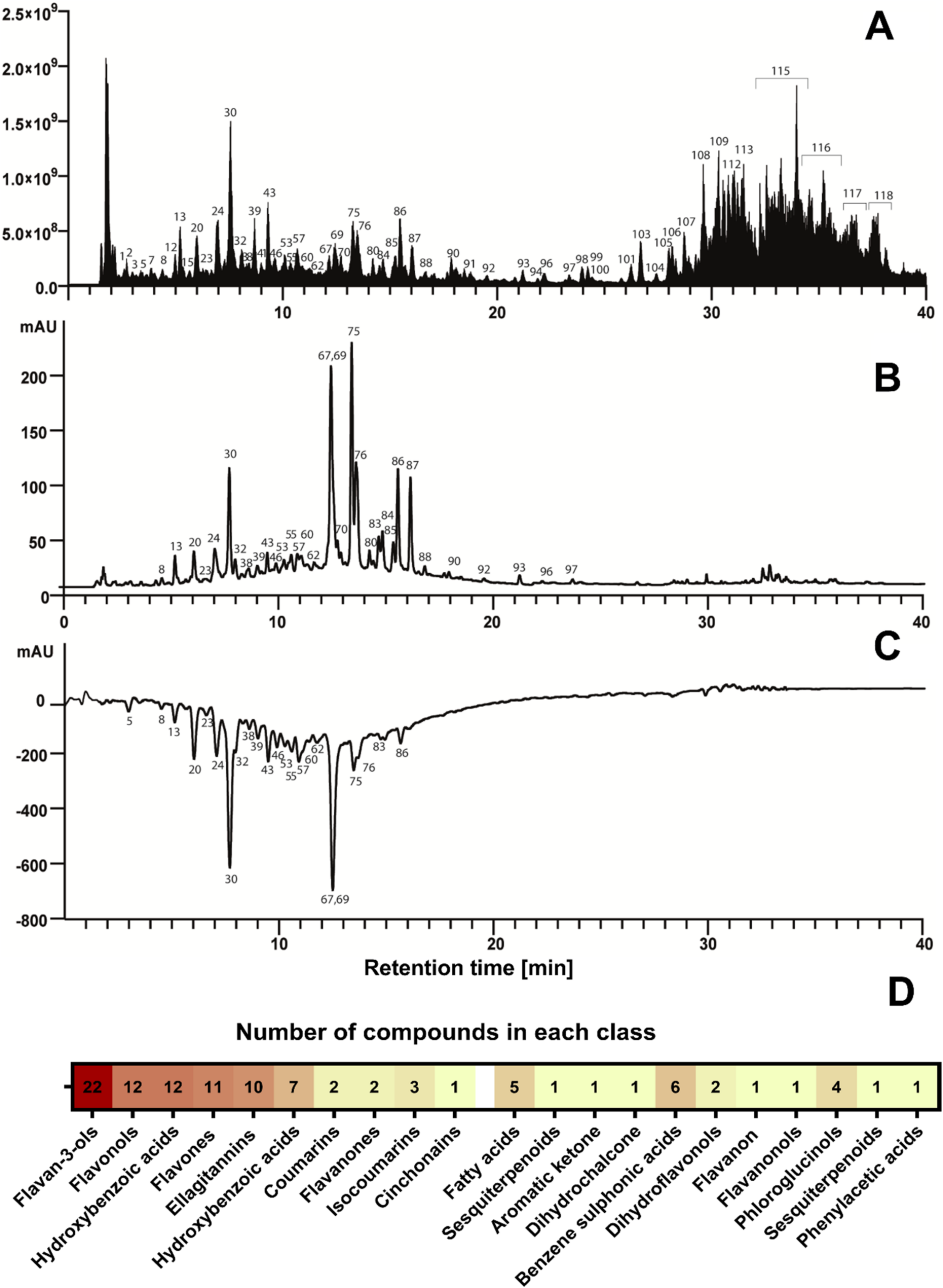
The antioxidant characteristics and chromatographic profiles of the phenolic components in AE extract. Panel **A**: the chromatogram obtained from LC-Q-Orbitrap HRMS analysis (black HRMS chromatograms). Panel **B**: shows the chromatogram from HPLC-DAD at 270 nm for the AE extract. Panel **C**: presents the antioxidant profile of the extract, as determined after post-column derivatization using the ABTS reagent, measured at 734 nm. For details on the peak identities, refer to Table 1. Panel **D**: the heat map illustrating the abundance of annotated compounds belonging to different classes. The numbers on the peaks correspond to the identified compounds listed in Table 1

Identification of phenolic constituents in the ethanol extract of the AE aerial part was carried out using the advanced LC-Q-Orbitrap HRMS chromatographic technique. A diverse array of phenolic compounds belonging to different groups were identified, emphasizing the plant’s potential therapeutic properties. Following a detailed analysis of the UV-visible spectrum, 112 compounds were annotated, and each compound was categorized into specific groups (Fig. 3; Table 1). The compounds further were identified by examining the major m/z signals recorded in negative ion mode, alongside retention time and literature data, utilizing both full scan MS and MS2 spectra (Table 1). The analysis revealed a significant presence of flavan-3-ols, the most abundant group, with 22 distinct compounds including multiple forms of procyanidin (dimer, trimer, tetramer, and pentamer) (Fig. 3D). The next most prevalent groups were hydroxycinnamic acids (HCA) and flavonols, with 12 identified compounds in each. Ellagitannins and flavones also showed notable diversity, with 10 and 11 compounds respectively. Other identified groups, such as hydroxybenzoic acids, coumarins, flavanones, isocoumarins, etc. are represented by a smaller number of compounds in the extract. The.

**Table 1:** Phenolic compounds identified in *A. eupatoria* plant extracts in negative polarity with retention time (RT) and relevant MS and MS/MS information

No	Class of compounds	Tentative Identification	RT (Min)	Molecular Formula	Molecular Weight	[M-H] Theoretical m/z)	[M-H] Observed (m/z)	Mass Error (ppm)	Main Product fragments (m/z)
1	HBA	Galloyl glucoside	2.72	C₁₃H₁₆O₁₀	332.07435	331.066525	331.067075	−1.661297529	169,01; 125,02
2	HCA	Syringoylquinic acid	2.74	C₁₆H₂₀O₁₀	372.10565	371.097825	371.098385	−1.509036061	191,06; 173,04; 135,04
3	HBA	Galloylquinic acid	2.85	C₁₄H₁₆O₁₀	344.07435	343.066525	343.067105	−1.6906342	191.06
4	HBA	Protocatechuic acid glucoside	2.99	C₁₃H₁₆O₉	316.079435	315.07161	315.072295	−2.174108927	153,02; 109,03
5	Flavan-3-ols	Procyanidin trimer	3.33	C₄₅H₃₈O₁₈	866.20582	865.197995	865.199215	−1.410081862	287,06; 575,12; 695,14
6	HBA	Galloyl glucoside	3.64	C₁₃H₁₆O₁₀	332.07435	331.066525	331.067075	−1.661297529	169,01; 125,02
7	HBA	Vanillic acid glucoside	3.87	C₁₄H₁₈O₉	330.095085	329.08726	329.087825	−1.716869866	167,03; 108,02; 152,01
8	Flavan-3-ols	Epi/Catechin glucoside	4.38	C₂₁H₂₄O₁₁	452.131865	451.12404	451.124755	−1.584929945	289,07; 245,08
9	HCA	Dihydroferulic acid glucuronide	4.39	C₁₆H₂₀O₁₀	372.10565	371.097825	371.098435	−1.643771423	167,03; 125,02
10	Ellagitanins	Pedunculagin I isomer	4.54	C₃₄H₂₄O₂₂	784.07593	783.068105	783.069645	−1.966623325	301,00; 481,06; 275,02
11	Ellagitanins	Sanguiin H-4 isomer	4.72	C₂₇H₂₂O₁₈	634.08062	633.072795	633.074035	−1.9587005	301,00; 275,02
12	Flavanols	Epi/Catechin glucoside	4.95	C₂₁H₂₄O₁₁	452.131865	451.12404	451.124755	−1.584929945	289,07; 167,11; 137,02
13	HCA	3-O-Caffeoylquinic acid	5.22	C₁₆H₁₈O₉	354.095085	353.08726	353.087785	−1.486884573	191,06; 135,04; 179,03
14	HCA	p-Coumaroyl glucoside	5.40	C₁₅H₁₈O₈	326.10017	325.092345	325.092935	−1.814868941	163,04; 119,05
15	Flavan-3-ols	Epi/Catechin glucoside	5.60	C₂₁H₂₄O₁₁	452.131865	451.12404	451.124935	−1.983933288	289,07; 137,02
16	HCA	Caffeoyl glucoside	5.66	C₁₅H₁₈O₉	342.095085	341.08726	341.087835	−1.685785626	179,03; 161,02
17	Ellagitanins	Sanguiin H-4 isomer	5.71	C₂₇H₂₂O₁₈	634.08062	633.072795	633.074045	−1.974496472	301,00; 275,02
18	Flavonols	Quercetin diglucoside	5.96	C₂₇H₃₀O₁₇	626.148305	625.14048	625.141415	−1.495663822	463,03; 301,04
19	Ellagitanins	Pedunculagin I isomer	5.96	C₃₄H₂₄O₂₂	784.07593	783.068105	783.069395	−1.647366291	301,00; 481,06; 275,02
20	Flavan-3-ols	Procyanidin dimer	6.00	C₃₀H₂₆O₁₂	578.14243	577.134605	577.135455	−1.472793336	289,07; 407,08; 125,02
21	Coumarins	Esculin	6.27	C₁₅H₁₆O₉	340.079435	339.07161	339.072125	−1.5188532	177.02
22	Dihydroflavonols	Taxifolin glucoside	6.49	C₂₁H₂₂O₁₂	466.11113	465.103305	465.103975	−1.440540183	285,04; 151,00
23	Flavan-3-ols	Procyanidin tetramer	6.59	C₆₀H₅₀O₂₄	1154.26921	1153.261385	1153.263145	−1.526106764	575,12; 863,18; 287,06
24	Flavan-3-ols	Procyanidin dimer	6.99	C₃₀H₂₆O₁₂	578.14243	577.134605	577.135565	−1.66339012	289,07; 407,08; 125,02
25	HCA	p-Coumaroylquinic acid	7.03	C₁₆H₁₈O₈	338.10017	337.092345	337.092905	−1.661265847	163,04; 119,05; 191,06
26	Ellagitanins	Sanguiin H-4 isomer	7.09	C₂₇H₂₂O₁₆	634.08062	633.072795	633.073935	−1.800740782	301,00; 463,05; 275,02
27	HCA	5-O-Caffeoylquinic acid	7.30	C₁₆H₁₈O₉	354.095085	353.08726	353.087765	−1.430241352	191,06; 173,04
28	Flavan-3-ols	Procyanidin trimer	7.43	C₄₅H₃₈O₁₈	866.20582	865.197995	865.199285	−1.490988198	287,06; 577,14; 425,09
29	HCA	4-O-Caffeoylquinic acid	7.47	C₁₆H₁₈O₉	354.095085	353.08726	353.087775	−1.458562962	135,04; 173,04; 191,06
30	Flavan-3-ols	Catechin	7.57	C₁₅H₁₄O₆	290.07904	289.071215	289.071775	−1.937238891	245,08; 205;05
31	HCA	Feruloylquinic acid	7.73	C₁₇H₂₀O₉	368.110735	367.10291	367.103365	−1.239434468	134,04; 193,05
32	Flavan-3-ols	Procyanidin tetramer	7.73	C₆₀H₅₀O₂₄	1154.26921	1153.261385	1153.263115	−1.50009358	575,12; 865,20; 287,06
33	Isocoumarins	Brevifolincarboxylic acid	7.86	C₁₃H₈O₈	292.02192	291.014095	291.014675	−1.993030612	247,02; 191,03; 219,03
34	Flavan-3-ols	Procyanidin pentamer	8.02	C₇₅H₆₂O₃₀	1442.3326	1441.324775	1441.327045	−1.574939971	1153,26; 865,20; 287,06
35	Flavonols	Quercetin rutinoside	8.07	C₂₇H₃₀O₁₆	610.15339	609.145565	609.146545	−1.608810859	301,04; 463,09; 447,09
37	Flavan-3-ols	Procyanidin dimer	8.32	C₃₀H₂₆O₁₂	578.14243	577.134605	577.135625	−1.767352003	289,07; 407,08; 125,02
38	Flavan-3-ols	Procyanidin dimer	8.45	C₃₀H₂₆O₁₂	578.14243	577.134605	577.135615	−1.750025022	289,07; 407,08; 125,02
39	Sesquiterpenoids	Roseoside	8.69	C₁₉H₃₀O₈	386.19407	385.186245	385.186805	−1.453842154	153,09; 205,12; 71,01
40	Isocoumarins	Brevifolincarboxylic acid	8.86	C₁₃H₈O₈	292.02192	291.014095	291.014675	−1.993030612	175,04; 247,02; 147,04
41	HCA	p-Coumaroylquinic acid	9.00	C₁₆H₁₈O₈	338.10017	337.092345	337.092925	−1.72059677	163,04; 119,05; 93,03
42	Isocoumarins	Brevifolin	9.24	C₁₂H₈O₆	248.03209	247.024265	247.024445	−0.728673355	191,03; 247,02; 219,03
43	Flavan-3-ols	Epicatechin	9.31	C₁₅H₁₄O₆	290.07904	289.071215	289.071775	−1.937238891	245,07; 205,05
44	Flavan-3-ols	Procyanidin haxamer	9.49	C₉₀H₇₄O₃₆	1730.39599	1729.388165	1729.391375	−1.856147778	865,21; 575,12; 287,06
45	Ellagitanins	Agrimonic acid A or B	9.53	C₄₈H₃₂O₃₁	1104.092765	1103.08494	1103.087055	−1.917350082	935,08; 801,08; 633,06
46	HCA	p-Coumaroylquinic acid	9.64	C₁₆H₁₈O₈	338.10017	337.092345	337.092925	−1.72059677	163,044;119,05; 93,03
47	Flavan-3-ols	Procyanidin dimer	9.66	C₃₀H₂₆O₁₂	578.14243	577.134605	577.135495	−1.542101257	289,07; 407,08; 125,02
48	Flavanon	Eriodictyol glucoside	9.68	C₂₁H₂₂O₁₁	450.116215	449.10839	449.109105	−1.592043293	259,06; 269,05; 287,06
49	Flavan-3-ols	Procyanidin trimer	9.84	C₄₅H₃₈O₁₈	866.20582	865.197995	865.199345	−1.560336487	287,06; 577,14; 425,09
51	Ellagitanins	Laevigatin F isomer	9.93	C₆₈H₄₈O₄₄	1568.15186	1567.144035	1567.146745	−1.729260323	935,08; 633,07; 301,00
52	Flavan-3-ols	Procyanidin tetramer	10.00	C₆₀H₅₀O₂₄	1154.26921	1153.261385	1153.263565	−1.890291333	865,20; 575,12; 287,06
53	Dihydroflavonols	Taxifolin glucoside	10.08	C₂₁H₂₂O₁₂	466.11113	465.103305	465.103765	−0.989027588	285,04; 151,00
54	Flavan-3-ols	Procyanidin tetramer	10.15	C₆₀H₅₀O₂₄	1154.26921	1153.261385	1153.263565	−1.890291333	865,20; 575,12; 287,06
55	Lignan	Pinoresinol	10.34	C₁₆H₂₂O₉	358.126385	357.11856	357.119115	−1.554105729	151,08; 177,06
56	Ellagitanins	Laevigatin F isomer	10.36	C₆₈H₄₈O₄₄	1568.15186	1567.144035	1567.146745	−1.729260323	935,08; 633,07; 301,00
57	Coumarins	Esculin	10.67	C₁₅H₁₆O₉	340.079435	339.07161	339.071905	−0.870022707	159,03; 177,04
57	Flavan-3-ols	Procyanidin dimer	10.68	C₃₀H₂₆O₁₂	578.14243	577.134605	577.135365	−1.316850512	289,07; 407,08; 125,02
59	Flavanonols	Taxifolin glucoside	10.87	C₂₁H₂₂O₁₂	466.11113	465.103305	465.103775	−1.010528188	285,04; 151,00
60	Flavonols	Quercetin-xylosyl-rutinoside	10.92	C₃₂H₃₈O₂₀	742.19565	741.187825	741.188965	−1.538071676	300,03; 591,14
61	Flavan-3-ols	Procyanidin tetramer	11.10	C₆₀H₅₀O₂₄	1154.26921	1153.261385	1153.263565	−1.890291333	575,12; 865,20; 287,06
62	Flavan-3-ols	Procyanidin pentamer	11.33	C₇₅H₆₂O₃₀	1442.3326	1441.324775	1441.327045	−1.574939971	1153,26; 865,20; 287,06
64	Isocoumarins	Methyl brevifolincarboxylate	11.67	C₁₄H₁₀O₈	306.03757	305.029745	305.030335	−1.934237594	217,01; 245,01; 273,04
65	Flavonols	Quercetin-glucosyl-xyloside	12.02	C₂₆H₂₈O₁₆	596.13774	595.129915	595.130865	−1.596290114	300,21; 301,03;
66	Flavanones	Eriodictyol-glucoside	12.14	C₂₁H₂₂O₁₁	450.116215	449.10839	449.109045	−1.458445254	269,05; 151,00; 179,00
67	Ellagitanins	Agrimoniin	12.19	C₈₂H₅₄O₅₂	1870.15813	1869.150305	1869.152965	−1.423106527	300,99; 935,08; 897,04
68	Ellagitanins	Ellagic acid pentoside	12.28	C₁₉H₁₄O₁₂	434.04853	433.040705	433.041445	−1.708846285	301,00; 271,06; 313,07
69	Flavonols	Quercetin-rutinoside	12.45	C₂₇H₃₀O₁₆	610.15339	609.145565	609.146315	−1.231232801	300,00; 302,04; 343,05
70	Flavones	Apigenin-glucoside (vitexin)	12.72	C₂₁H₂₀O₁₀	432.10565	431.097825	431.098455	−1.461385244	311,06; 283,06;341,07
71	Flavanones	Eriodictyol-glucoside	12.73	C₂₁H₂₂O₁₁	450.116215	449.10839	449.109045	−1.458445254	269,05; 179,00; 151,00
72	Flavones	Scutellarin	12.80	C₂₁H₁₈O₁₂	462.07983	461.072005	461.072835	−1.800152668	285
73	Flavan-3-ols	Procyanidin dimer	12.97	C₃₀H₂₆O₁₂	578.14243	577.134605	577.135435	−1.438139375	289,70; 125,02; 407,08
74	HBA	Ellagic acid	13.18	C₁₄H₆O₈	302.00627	300.998445	300.998875	−1.428578809	229,01; 257,01
75	Flavonols	Quercetin-hexoside	13.29	C₂₁H₂₀O₁₂	464.09548	463.087655	463.088285	−1.360433588	300,03; 386,04
76	Flavonols	Quercetin-hexoside	13.47	C₂₁H₂₀O₁₂	464.09548	463.087655	463.088285	−1.360433588	300.03
77	Flavones	Luteolin-apiosyl-glucoside	13.67	C₂₆H₂₈O₁₅	580.142825	579.135	579.135805	−1.390004058	284,03; 447,09; 579,14
79		Unknown	14.03	C₂₀H₃₂O₉	416.204635	415.19681	415.197635	−1.987009486	292,87; 306,90; 89,02
80	Flavones	Kaempferol-rutinoside	14.20	C₂₇H₃₀O₁₅	594.158475	593.15065	593.151725	−1.812355765	285,04; 482,12; 327,05
81	Flavonols	Isorhamnetin-hexoside	14.27	C₂₂H₂₂O₁₂	478.11113	477.103305	477.103975	−1.404308025	315,07; 477,10; 161,02
82	Flavonols	Isorhamnetin-glucoside-rhamnoside	14.47	C₂₈H₃₂O₁₆	624.16904	623.161215	623.162175	−1.540532332	315.05
83	Flavonols	Quercetin-malonyl-hexoside	14.52	C₂₄H₂₂O₁₅	550.095875	549.08805	549.088975	−1.684611421	300,03; 505,10; 271,02
84	Flavones	Luteolin-apiosyl-malonyl-glucoside	14.93	C₂₉H₃₀O₁₈	666.14322	665.135395	665.136715	−1.984558347	284,03; 489,10; 579,14
85	Flavones	Kaempferol-glucoside	15.25	C₂₁H₂₀O₁₁	448.100565	447.09274	447.093415	−1.509753883	284,03; 285,03; 327,05
86	Flavonols	Quercetin-rhamnoside	15.46	C₂₁H₂₀O₁₁	448.100565	447.09274	447.093385	−1.44265371	300,03; 301,03; 283,03
87	Flavones	Apigenin-glucuronide	16.02	C₂₁H₁₈O₁₁	446.084915	445.07709	445.077755	−1.494123187	269.05
88	Flavonols	Kaempferol-malonyl-hexoside	16.68	C₂₄H₂₂O₁₄	534.10096	533.093135	533.094055	−1.725777242	285,04; 284,03; 489,10
89	Dihydrochalcone	Phloridzin	16.94	C₂₁H₂₄O₁₀	436.13695	435.129125	435.129825	−1.608717872	167,03; 273,08; 229,08
90		Unknown	17.86	C₂₂H₂₄O₁₀	448.13695	447.129125	447.129745	−1.386624054	145,03; 307,08; 139,04
91	Aromatic ketone	C-Veratroylglycol	18.09	C₁₀H₁₂O₅	212.068475	211.06065	211.060365	1.350322763	151,04;109,03; 165,05
92	Flavones	Kaempferol-acetyl-glucoside	19.49	C₂₃H₂₂O₁₂	490.11113	489.103305	489.104065	−1.553863963	284,03; 285,04; 255,02
93	Flavones	Tiliroside isomer	21.22	C₃₀H₂₆O₁₃	594.137345	593.12952	593.130425	−1.525805021	285,04; 284,03; 447,09
94	Flavones	Tiliroside isomer	21.90	C₃₀H₂₆O₁₃	594.137345	593.12952	593.130435	−1.542664745	285,04; 284,03; 447,09
95	Flavonols	Quercetin	22.12	C₁₅H₁₀O₇	302.042655	301.03483	301.035275	−1.478234263	151,00; 179,00; 107,01
96	Phenylacetic acids	Homoveratric acid	22.18	C₁₀H₁₂O₄	196.07356	195.065735	195.065345	1.999326022	95,05;79,02; 151,08
97	HCA	Tricoumaroyl spermidine	23.36	C₃₄H₃₇O₆N₃	583.268237	582.260412	582.261215	−1.379108013	342,14; 462,20; 119,05
98		Unknown	23.96	C₃₇H₆₀O₁₂	696.40848	695.400655	695.401675	−1.466780327	487, 34; 207,05; 649,40
99	Fatty acids	Oxo-dihydroxy-octadecenoic acid isomer	24.22	C₁₈H₃₂O₅	328.224975	327.21715	327.217595	−1.359953169	171,10; 211,13; 229,14
100	Fatty acids	Oxo-dihydroxy-octadecenoic acid isomer	24.43	C₁₈H₃₂O₅	328.224975	327.21715	327.217595	−1.359953169	211,13; 171,10; 229,14
101	Fatty acids	Trihydroxy-octadecenoic acid isomer	26.25	C₁₈H₃₄O₅	330.240625	329.2328	329.233215	−1.260506244	211,13; 229,14; 183,14
102	Flavones	Kaempferol	26.66	C₁₅H₁₀O₆	286.04774	285.039915	285.040225	−1.087566982	285,04; 181,90
103	Fatty acids	Traumatic acid isomer	26.68	C₁₂H₂₀O₄	228.13616	227.128335	227.128235	0.440279721	183,14; 184,18; 165,47
104	Fatty acids	Traumatic acid isomer	27.04	C₁₂H₂₀O₄	228.13616	227.128335	227.128235	0.440279721	183,14; 165,12; 200,21
105		Unknown	28.02	C₁₂H₁₆O₆	256.09469	255.086865	255.087015	−0.588034982	195,07; 177,06; 151,00
106	Cinchonains	Phylloflavan	28.14	C₂₆H₂₆O₁₀	498.1526	497.144775	497.145635	−1.729878384	289,07; 301,07; 215,07
107		Unknown	28.72	C₁₃H₂₀O₄	240.13616	239.128335	239.128435	−0.41818549	193,06; 221,08; 179,08
108	HBA	Olivetolic acid	29.62	C₁₂H₁₆O₄	224.10486	223.097035	223.096805	1.030941536	109,06; 123,08; 179,11
109	Benzene sulphonic acids	Undecylbenzenesulphonic acid	30.34	C₁₇H₂₈O₃S	312.175917	311.168092	311.168615	−1.680763592	183,01; 197,03
110	Benzene sulphonic acids	Undecylbenzenesulphonic acid	30.53	C₁₇H₂₈O₃S	312.175917	311.168092	311.168605	−1.648626621	183,01; 198,04
111	Benzene sulphonic acids	Undecylbenzenesulphonic acid	30.61	C₁₇H₂₈O₃S	312.175917	311.168092	311.168615	−1.680763592	183,01; 198,04
112	Benzene sulphonic acids	Dodecylbenzenesulfonic acid	30.79	C₁₀H₃₀O₃S	326.191567	325.183742	325.184205	−1.423810419	183,01; 197,03
113	Benzene sulphonic acids	Dodecylbenzenesulfonic acid	31.00	C₁₈H₃₀O₃S	326.191567	325.183742	325.184205	−1.423810419	183,01; 325,18; 197,03
114	Benzene sulphonic acids	Tridecylbenzenesulfonic acid	31.50	C₁₉H₃₂O₃S	340.207217	339.199392	339.199705	−0.922761088	183,01; 170,00; 197,03
115	Phloroglucinols	Phloraspine	33.98	C₂₃H₂₈O₈	432.17842	431.170595	431.171115	−1.206019163	207,07; 223,10; 195,06
116	Phloroglucinols	Desaspidin/Flavaspidic acid	35.21	C₂₄H₃₀O₈	446.19407	445.186245	445.187135	−1.999163294	237,11; 239,12; 207,07
117	Phloroglucinols	Aspidin	36.52	C₂₅H₃₂O₈	460.20972	459.201895	459.202725	−1.80748383	223,10; 235,10
118	Phloroglucinols	6-[(3-butanoyl-2,6-dihydroxy-4-methoxy-5-methylphenyl)methyl]−3,5-dihydroxy-4,6-dimethyl-2-(2-methylbutanoyl)cyclohexa-2,4-dien-1-one	37.57	C₂₆H₃₄O₈	474.22537	473.217545	473.218235	−1.458103165	237,11; 235,09

*HBA hydroxybenzoic acids and derivatives, *HCA* hydroxycinnamic acids and derivatives

HPLC analysis followed by post-column derivatization with ABTS reagent was performed to reveal the list of compounds in the AE extract that possess antioxidant properties. This reaction caused a noticeable shift in the UV-visible spectrum by altering the absorption characteristics of the ABTS reagent, evident as discoloration. Negative peaks at 734 nm in the chromatogram indicated the presence of antioxidants in the eluate (Fig. 3C). Prior to derivatization, the DAD detector identified approximately 30 major compounds at 270 nm in the AE extract chromatograms (Fig. 2B), of which 23 compounds across 8 different groups demonstrated antioxidant activity after derivatization. Ten of the flavan-3-ols out of 22 expressed antiradical activity, significantly contributing to the extract’s antiradical capacity. The second major group was flavonols, which include six compounds with antioxidant properties. The antioxidant properties of these major phenolic compounds have also been documented in the literature as well (Table 3).

### Morphological analysis of apoptosis by Hoechst 33258 staining

**Fig. 4:**
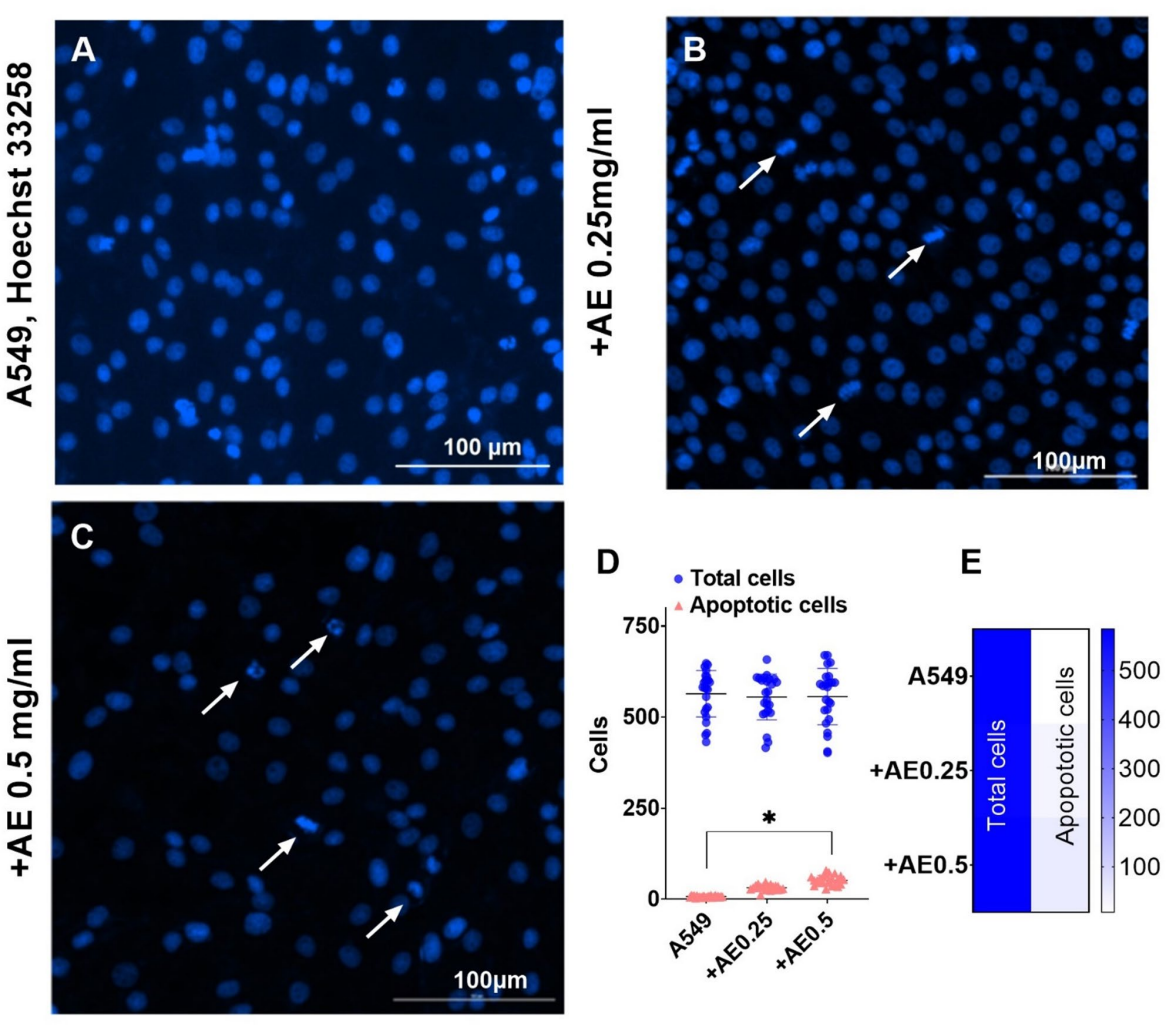
Assessment of apoptosis by Hoechst 33,258 staining (blue) in A549 cells. Arrowheads indicate apoptotic cell nuclei. **A** Control cell nuclei have smooth edges. **B** Treatment of cells with AE extract at 0.25 mg/mL (**C**) and 0.5 mg/mL induced chromatin condensation, nuclear shrinkage, and fragmentation. **D** The rate of apoptotic cells was detected by morphological analysis using Hoechst 33,258 staining. **E** Heatmap illustrating the apoptotic rate of A549 cells under different concentrations of AE extract compared to control, *p* < 0.05. Results represent means ± SD from at least three independent experiments

Hoechst 33258 stained apoptotic cells can be morphologically distinguished from viable cells by karyopyknosis, rough edges of their nuclei, and signs of nuclear fragmentation (Fig. 4, marked with arrows). All treatment variants induced an elevation of apoptosis rate in A549 cells compared to untreated control (Fig. 4). In control cells, the apoptosis rate (mean) was 1.3%, while 24 h incubation with AE 0.25 mg/mL increased the rate of apoptotic cells to 5.8% (p - ns; Fig. 4, B, D, and E). Treatments with AE 0.5 mg/mL increased the rates of apoptotic cells up to 9.6% (*p* < 0.05; Fig. 4, C, D, and E).

### AE extract modulates the PI3K/Akt/mTOR signaling pathway

The PI3K/Akt/mTOR signaling pathway plays a critical role in a wide range of cancer types, contributing to processes such as abnormal cell proliferation, dysregulated hypoxic responses, metastasis, and inflammation. Based on previous investigations in cancer models and the aggressive nature of lung adenocarcinoma, this study focused on the A549 human non-small cell lung carcinoma (NSCLC) cell line as the experimental model [30]. This cell line was chosen based on evidence of the role of downregulation of the PI3K/Akt pathway in A549 programmed cell death [36]. The main objective was to investigate the effect of the ethanolic extract of *A. eupatoria* on the PI3K signaling axis and to identify, through in silico docking analysis, potential phytochemicals from AE with high affinity toward components of the PI3K pathway. Initial cytotoxicity assays revealed that the AE extract exhibits a moderate cytotoxic effect on A549 cells, with a working concentration of 0.5 mg/mL used for mechanistic studies. While AE did not demonstrate strong cytotoxicity in vitro, previous in vivo studies for other plant extracts (*Hypericum alpestre* and *Rumex obtusifolius*) by our group showed pronounced tumor size reduction with similar plant-based treatments lacking direct cytotoxicity [24, 26]. This prompted further investigation into whether AE exerts anti-cancer effects through modulation of signaling pathways.

**Fig. 5:**
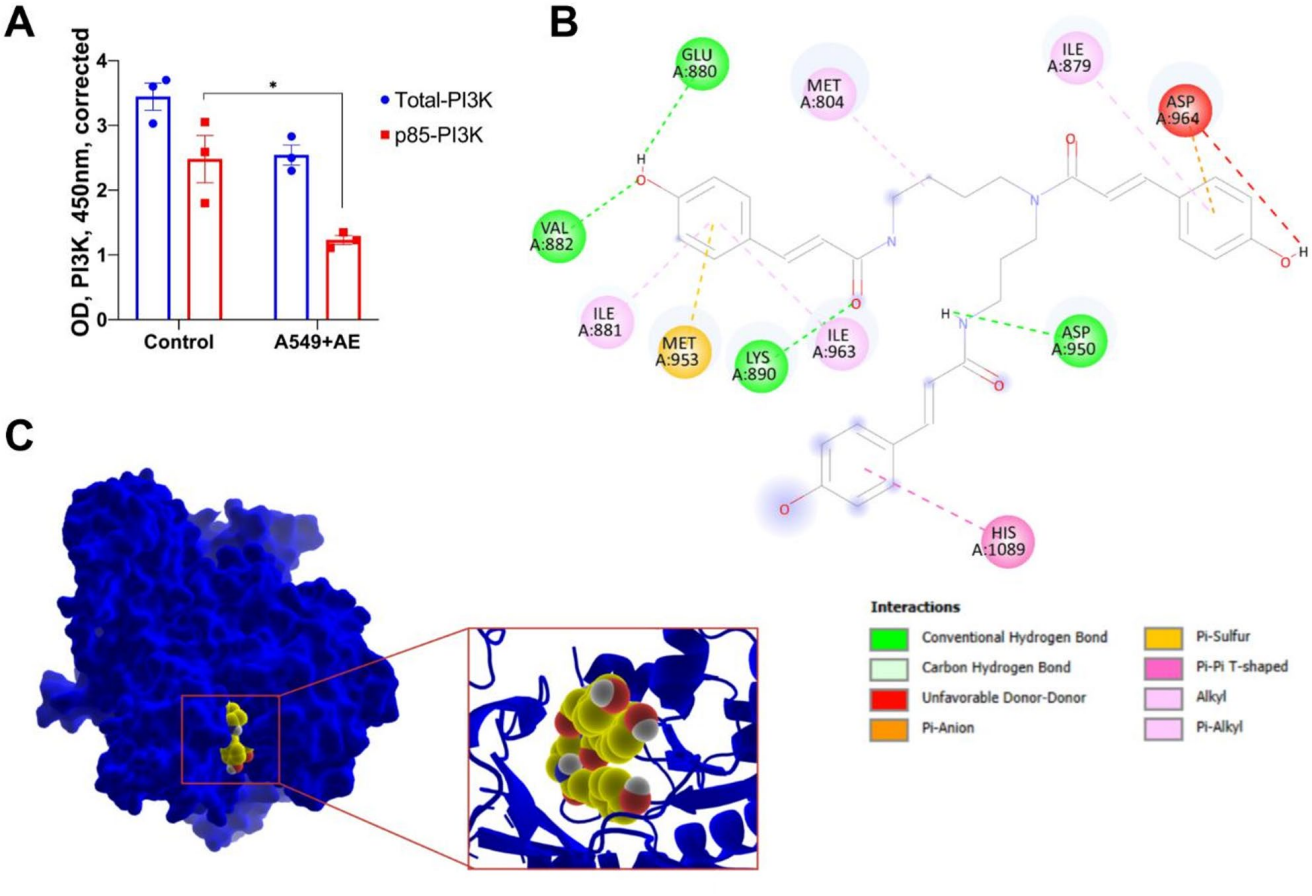
**A** - In vitro regulatory effect of *A. eupatoria* ethanol extract on total and phosphorylated PI3K quantitative changes in A549 cells (*n* = 3, * - *p* ≤ 0.05). **B** - In silico 2D binding analysis and interaction types of tricoumaroyl spermidine on Pi3K. **C** – In silico 3D Visualization of the interaction of the binding pocket of Pi3K with tricoumaroyl spermidine marked in green. Results represent means ± SD from at least three independent experiments

The effect of AE extract on the levels of phospho-PI 3 kinase p85 and total PI3K was measured on A549 cells. Based on the obtained data, AE extract appears to have significant inhibitory effects on PI3K activity in A549 lung adenocarcinoma cells (Fig. 5, A). The extract reduces the total amount of PI3K by approximately 30%. This suggests that either the herb’s compounds affect the gene expression or proteins involved in PI3K synthesis, or they directly interact with PI3K to inhibit its production. The extract also reduces the quantity of phosphorylated (activated) PI3K by about 50%. Phosphorylation is a crucial step in activating PI3K, and inhibiting this process can lead to deviations in the PI3K metabolic pathway. The observed effects indicate that the compounds present in common agrimony may influence both gene expression and protein regulation involved in PI3K synthesis. Additionally, they may directly interact with PI3K to inhibit its phosphorylation, thereby preventing its activation.

**Fig. 6:**
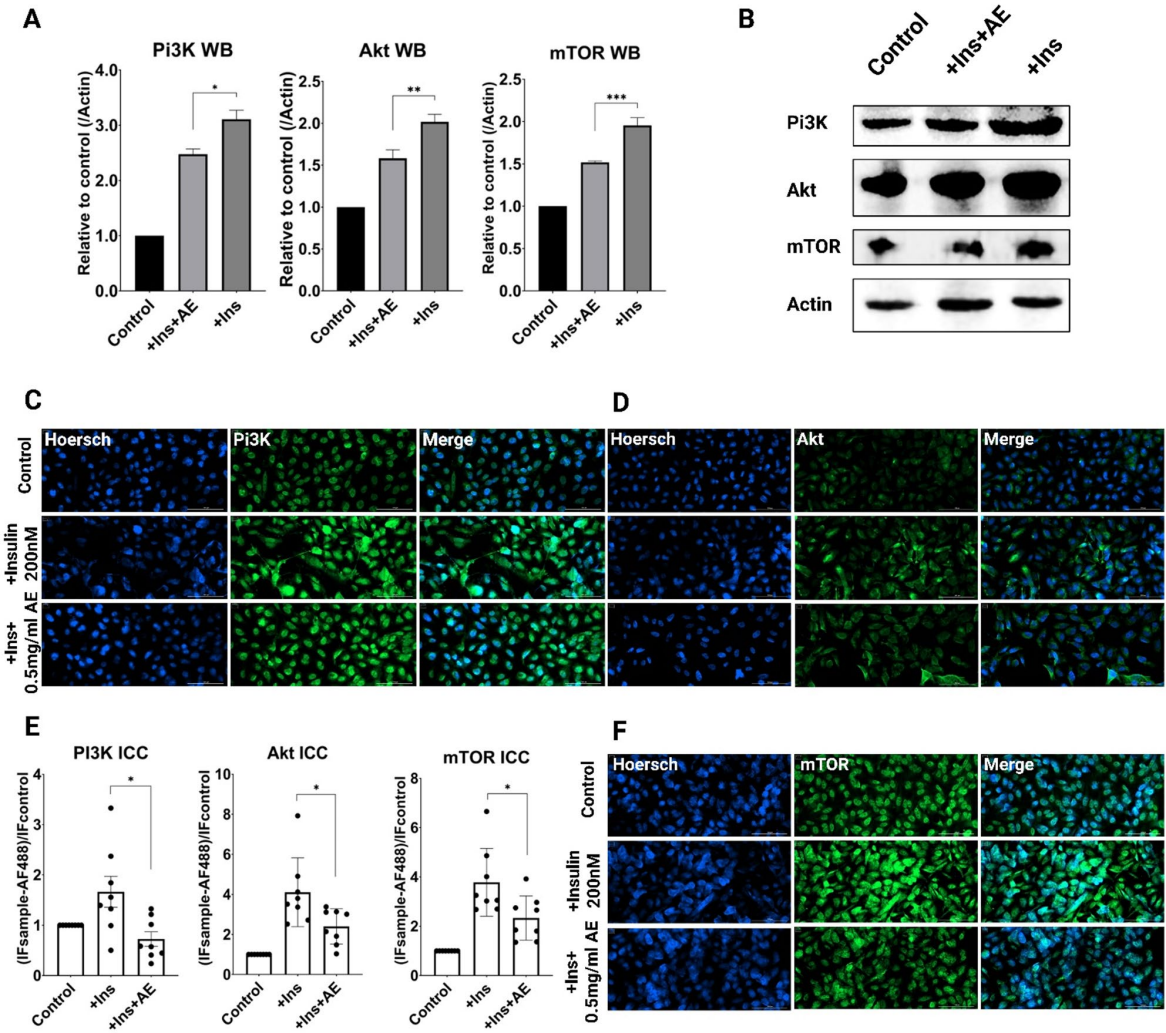
Suppression of the PI3K/Akt/mTOR pathway in the Insulin-induced A549 cells by *A. eupatoria* extract. **A**. Quantitative densitometric graphs of PI3K, Akt, and mTOR expression levels following Insulin stimulation, with plant extract treatment. Protein levels were normalized to β-actin. Data represent mean ± SD (*n* = 3 independent experiments); one-way ANOVA with Tukey’s multiple comparisons post-hoc test. **B**. Representative Western blot bands for PI3K, Akt, and mTOR under Insulin stimulation, with plant extract treatment. **C**, **D**, and **F**. Representative confocal ICC images of PI3K (**C**), Akt (**D**), and mTOR (**F**) expression. Scale bar = 100 μm; magnification 20×. **E**. Quantitative analysis of PI3K, Akt, and mTOR expression based on immunocytochemistry (ICC). Fluorescence intensities were background-corrected (Alexa Fluor 488 signal in untreated cells subtracted) and normalized to the control. Data represent mean ± SD (*n* = 3 independent experiments; 8 fields per condition per experiment); one-way ANOVA with Tukey’s multiple comparisons post-hoc test

To activate the PI3K/Akt/mTOR pathway, cells were treated with insulin, a known upstream stimulator. Following insulin stimulation, AE extract was applied, and the effect on PI3K, Akt, and mTOR protein levels was assessed via Western blot (WB) and immunocytochemistry/immunofluorescence (ICC/IF). Western blot analysis (Figure X, A and B) revealed that AE at 0.5 mg/mL reduced insulin-stimulated PI3K levels by approximately 20% (*p* ≤ 0.05), Akt by 22% (*p* ≤ 0.01), and mTOR by 25% (*p* ≤ 0.001). ICC/IF analysis (Fig. 6, C–F) confirmed these findings. Insulin treatment significantly increased fluorescence intensity of PI3K/Akt/mTOR components (2–4-fold increase; *p* ≤ 0.001), whereas AE extract reduced PI3K intensity by approximately 2.3-fold (*p* ≤ 0.05), Akt by around 40% (*p* ≤ 0.05), and mTOR by 35% (*p* ≤ 0.05). Complementary ELISA data demonstrated a decrease in both total and phosphorylated PI3K levels following AE treatment, supporting WB and ICC results.

**Fig. 7:**
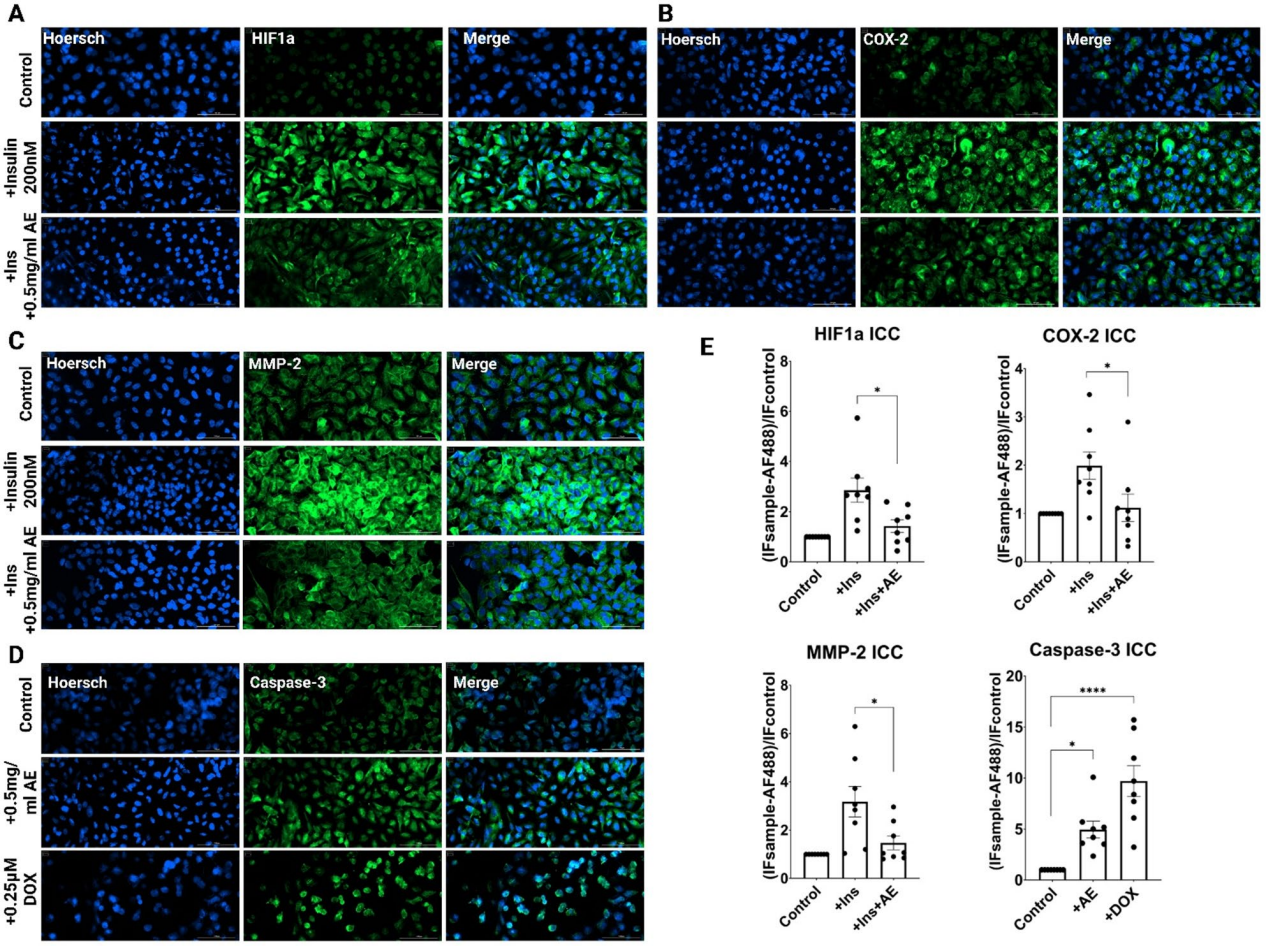
Immunofluorescence analysis of HIF-1α, MMP-2, COX-2, and Caspase-3 expression in insulin-stimulated A549 cells treated with *Agrimonia eupatoria* extract. **A**, **B**, **C**, and **D**. Representative confocal ICC images of HIF1a (A), COX-2 (B), MMP-2 (**C**), and Caspase-3 (**D**) expression. Scale bar = 100 μm; magnification 20×. **E**. Quantitative analysis of HIF1a, COX-2, MMP-2, and Caspase-3 expression based on immunocytochemistry (ICC). Fluorescence intensities were background-corrected (Alexa Fluor 488 signal in untreated cells subtracted) and normalized to the control. Data represent mean ± SD (*n* = 3 independent experiments; 8 fields per condition per experiment); one-way ANOVA with Tukey’s multiple comparisons post-hoc test

To further evaluate AE’s impact on PI3K-associated downstream processes, we examined HIF-1α, COX-2, and MMP-2 expression under insulin stimulation. These markers are tightly linked to hypoxia, inflammation, and metastasis, respectively. Insulin stimulation significantly upregulated all three markers. However, AE extract effectively inhibited their expression (Fig. 7, A–C and E): HIF-1α and MMP-2 levels were reduced by 3 and 5-fold (*p* ≤ 0.05), and COX-2 levels also showed a statistically significant decrease (*p* ≤ 0.05). Finally, Caspase-3 expression was evaluated to assess AE’s potential pro-apoptotic activity. As shown in Fig. 7, D and E, AE extract modestly enhanced Caspase-3 expression, with effects comparable to the positive control Doxorubicin (DOX), as confirmed via both ICC and chromatin condensation studies (Hoechst staining). These findings, alongside MTT data, suggest that AE can moderately induce apoptosis in A549 cells.

### Molecular docking and ADME

Molecular docking assessments were conducted using AutoDock Vina on the binding pocket defined by the crystal structure of PI3K complexed with a pyrrolopyridinone inhibitor which was used as a reference (PDB ID: 6C1S) [33]. This analysis included redocking the e-reference inhibitor alongside 33 compounds identified in the extract, primarily focusing on their binding affinities.

The major compounds of the extract were marked for computational docking studies based on peak intensity of the MS and UV chromatograms (Fig. 3A and B). Pharmacokinetic properties, such as molecular weight (MW), number of hydrogen bond acceptors (HBA), number of hydrogen bond donors (HBD), and the octanol-water partition coefficient (logP), were computationally determined for each compound (Table 2). These properties were evaluated against Lipinski’s rule of five, which dictates acceptable thresholds for optimal drug-like characteristics [37].

**Table 2:** Molecular Docking analysis and ADME properties of major compounds from the AE extract

Ligand	Binding ffinity (kJ/mol)	MW Da	HBA	HBD	logP	Ro5 Viol.s
Quercetin Xylosyl Rutinoside	−10.3	742.63	20	12	−2.77	3
Agrimoniin	−9.9	1871.3	52	29	4.2	3
Taxifolin Glucoside	−9.7	466.39	12	8	−1.01	2
Procyanidin Trimer	−9.5	866.77	18	15	1.77	3
Tricoumaroyl Spermidine	−9.4	583.67	6	5	4	1
Quercetin Rutinoside	−9.4	610.52	16	10	−1.27	3
Apigenin Glucuronide	−9.2	446.4	11	6	0.2	2
Procyanidin Dimer	−9.2	578.52	12	10	1.53	3
Quercetin Rhamnoside	−9	448.38	11	7	0.62	2
Luteolin Apiosyl Malonyl Glucoside	−8.9	534.4	14	7	0.2	3
Reference	**−8.9**	376.4	7	0	1.2	0
Epicatechin Glucoside	−8.8	454.41	11	8	−1.08	2
Kaempferol Glucoside	−8.7	448.4	11	7	0.7	2
Kaempferol Rutinoside	−8.7	594.52	15	9	−0.73	3
Phylloflavan	−8.5	498.5	10	7	1.9	1
Catechin	−8.4	290.27	6	5	0.85	0
3-O-Caffeoylquinic Acid	−8.3	354.31	9	6	−0.38	1
Esculin	−8.2	340.28	9	5	−0.62	0
Kaempferol Acetyl Glucoside	−8.2	490.4	12	6	0.7	2
Tiliroside	−8	594.5	13	7	2.5	3
Epicatechin	−7.9	290.27	6	5	0.85	0
Pinoresinol	−7.8	358.39	6	2	2.26	0
Roseoside	−7.5	386.44	8	5	0.01	0
P-Coumaroylquinic Acid	−7.4	338.31	8	5	0.05	0
Aspidin	−7.3	460.5	8	4	4.1	0
Desaspidin	−7.3	446.5	8	5	3.8	0
Flavaspidic Acid	−7.3	446.5	8	5	3.8	0
Olivetolic Acid	−7.3	224.25	4	3	3.7	0
Agrimophol	−6.9	474.5	8	4	4.6	0
Phloraspine	−6.9	432.5	8	5	4.5	0
Traumatic Acid	−6.3	228.28	4	2	3	0
Trihydroxy Octadecenoic Acid	−6.1	330.5	5	4	3.1	0
Homoveratric Acid	−5.6	196.2	4	1	1	0

Results represent means ± SD from at least three independent experiments

* *MW* molecular weight, *HBA* number of hydrogen bond acceptors, *HBD* number of hydrogen bond donors, *logP* the octanol-water partition coefficient, *Ro5 Viol.s* Lipinski’s rule of five violations

Of the compounds studied, quercetin xylosyl rutinoside, agrimoniin, and taxifolin glucoside showed the highest binding affinities at −10.3, −9.9, and − 9.7 kJ/mol, respectively. However, all three compounds exceeded at least one criterion from Lipinski’s rule of five, with agrimoniin notably breaching three rules due to its high MW (1871.3) and extensive HBA and HBD counts.

Overall, among the 10 compounds exhibiting superior binding affinities than the reference, only tricoumaroyl spermidine maintained a minimal violation, breaching just one rule with an MW of 583.67 Da. The remaining compounds infringed upon the rule two or three times, indicating potential challenges in their drug development profiles due to pharmacokinetic concerns.

In contrast, of the 22 compounds with lower binding energies than the reference, a substantial fraction, specifically 14, showed full compliance with Lipinski’s rule of five. For instance, compounds such as catechin and pinoresinol with binding energies of −8.4 and − 7.8 kJ/mol respectively, displayed no violations, suggesting better suitability based on the rule’s criteria.

### Tricoumaroyl spermidine PI3K interaction analysis

In the computational analysis of the interactions between tricoumaroyl spermidine and the PI3K protein, detailed predictions illustrate the molecular basis of the binding affinity observed. The stable anchoring of tricoumaroyl spermidine within the ATP-competitive catalytic site of PI3K is facilitated by multiple hydrogen bonds. These key interactions primarily involve critical hinge region residues, namely ASP950 and GLU880, which are essential for securing ATP-competitive inhibitors and confirming the compound’s potential mechanism of action. Additional hydrogen bonds with ILE881 and ASP964 further enhance the interaction, facilitating a robust binding configuration. Moreover, tricoumaroyl spermidine engages in a variety of other non-covalent interactions that further stabilize its conformation within the protein. Notably, pi-anion interactions with ASP950 and pi-alkyl interactions with LYS890 and ILE963 indicate a dense network of contacts contributing to high binding affinity. Pi-sulfur interactions with MET953 and unfavorable donor-donor interactions, while typically less desirable, are also present and may influence the dynamic behavior of the ligand within the active site (Fig. 5B).

The 3D visualization of tricoumaroyl spermidine docked in the PI3K binding pocket illustrates a precise and optimal placement within the enzyme’s active site. The molecule is situated such that it achieves substantial surface complementarity with the contours of the binding pocket, suggesting a high degree of molecular interaction potential. The visualization indicates a snug fit, which is critical for effective inhibition of PI3K activity (Fig. 5C).

## Discussion

This study provides compelling evidence that *A. eupatoria* L extract exerts potent modulatory effects on the PI3K/Akt/mTOR signaling pathway and its downstream effectors, despite only moderate cytotoxicity in vitro in A548 cells. The findings support the hypothesis that certain plant-derived compounds may not directly kill cancer cells but can reprogram signaling networks, resulting in indirect anti-tumor effects such as reduced proliferation, inhibited metastasis, and enhanced apoptosis. Our results show that AE significantly downregulates PI3K, Akt, and mTOR expression, suggesting interference with one of the most central oncogenic pathways in NSCLC. Additionally, AE suppressed key hypoxia and metastasis-associated molecules, HIF-1α and MMP-2, both known to be transcriptional targets of the PI3K/Akt/mTOR axis. The downregulation of COX-2 further indicates potential anti-inflammatory effects of AE, which may have broader implications in the tumor microenvironment. Notably, the modest activation of Caspase-3 highlights AE’s potential to sensitize cancer cells to apoptosis, especially in combination with classical chemotherapeutics such as Doxorubicin. From a pharmacological perspective, these multi-target effects underscore AE’s potential as a polypharmacological agent, particularly valuable in complex diseases like cancer, where monotherapies often fail due to pathway crosstalk and redundancy.

For cellular analysis our experimental approach involved the selection of different cancer cell lines to answer specific biological questions. For the initial broad-spectrum cytotoxicity screening (MTT assay), a panel of diverse cell lines (HT-29, MCF-7, A549, HeLa) was used to assess the general cytotoxic potential of the AE extract across various tumor types. The cancer cell lines were selected based on literature demonstrating their reliance on dysregulated PI3K/Akt/mTOR signaling. Specifically, MCF-7 cells frequently harbor activating PIK3CA mutations [38], HT-29 cells exhibit constitutive PI3K pathway activation [39], HeLa cells possess highly active PI3K signaling due to HPV transformation [40], and A549 cells show EGFR-driven PI3K/Akt activity [41]. Cancer cell growth inhibiting properties of AE extract was also shown by other authors. For example, Ad’hiah et al. [4] demonstrated that AE extract has high inhibitory activity against tested rhabdomyosarcoma [RD] and HeLa cancer cells, whereas it was not cytotoxic against tested normal mouse embryonic fibroblast cells. For the subsequent in-depth mechanistic studies, we specifically focused on the A549 lung adenocarcinoma cell line. Although A549 cells demonstrated moderate resistance to direct cytotoxicity, they are a well-established model where the PI3K/Akt pathway is a critical driver of malignancy. This choice allowed us to investigate the extract’s ability to modulate key oncogenic pathways independent of high, immediate cell death, which was a central hypothesis of our study. Conversely, for the genotoxicity assessment, the highly sensitive HT-29 cell line was used to maximize the potential for detecting any DNA-damaging effects. An important consideration in interpreting of obtained findings is the relationship between the initiation of apoptosis and overall cell viability at the 24-hour time point. Our results show that treating A549 cells with the AE extract (0.25 and 0.5 mg/mL) for 24 h induced significant nuclear condensation (Fig. 4), a key morphological hallmark of apoptosis. However, under these identical conditions, the MTT assay did not detect a significant reduction in cell growth or viability (Fig. 2D).

This apparent discrepancy can be reconciled by considering the distinct biological endpoints measured by each assay. The MTT assay quantifies the overall metabolic activity of the cell population, serving as a proxy for cell viability. The lack of a significant decrease in the MTT signal at 24 h indicates that the bulk of the cell population had not yet undergone metabolic collapse or been eliminated. In contrast, Hoechst staining and Caspase-3 immunocytochemistry are sensitive detectors of the initiation of the apoptotic program. As apoptosis is a process that unfolds over a period, our results therefore suggest that at 24 h, the AE extract had successfully triggered pro-apoptotic signaling pathways in a fraction of the cells, leading to observable nuclear condensation and increased Caspase-3 expression. However, this early apoptotic state had not yet progressed to widespread cell death and the corresponding loss of metabolic function across the entire culture. Thus, the results are consistent, indicating that the extract’s primary effect at 24 h is pro-apoptotic and cytostatic, rather than overtly cytotoxic. The more pronounced cytotoxicity observed in the MTT assay at the 72-hour time point likely reflects the culmination of this initiated apoptotic process.

Our findings that AE extract can modulate the PI3K/Akt/mTOR pathway align with a growing body of evidence demonstrating the potential of phytochemicals as anticancer agents targeting this critical signaling axis [42]. A considerable number of plant-derived compounds have been identified as PI3K/Akt inhibitors, underscoring the value of natural products in cancer drug discovery. For instance, well-studied flavonoids like quercetin [43], polyphenols such as resveratrol from grapes [44], and curcumin from turmeric [45] have all been extensively documented to exert their anticancer effects, at least in part, by downregulating key components of the PI3K/Akt/mTOR pathway. The identification of tricoumaroyl spermidine as a potential inhibitor from *A. eupatoria* adds a novel compound to this list and reinforces the importance of exploring diverse plant sources for new therapeutic leads.

The investigation of the antioxidant properties of *A. eupatoria* extract underscores the complexity inherent in understanding the interactions between a plant extract’s chemical composition and its biological activities. AE is rich in flavonoids and phenolics, which are known for their antioxidant properties, and this fact is strongly evident in standard chemical assays where the extract demonstrated radical-scavenging activity. The HPLC analysis followed by post-column derivatization with ABTS reagent showed that out of 32 major compounds detected by the DAD detector in AE extract 22 possessed antiradical properties, which are also reported in the literature (Table 3). In contrast to this, the results from the cellular antioxidant activity (CAA) assay, which more closely mimics biological conditions, showed that AE displayed prooxidant properties within the cellular environment. This indicates that the extract’s effects can vary significantly under different testing conditions, reflecting the complexity of biological systems compared to in vitro models. High phenolic content and antioxidant activity of AE extract in different chemical tests were also shown in various studies [6, 7, 46, 47]. However, its dual effect was reported first time by us. This dual effect can be due to the phenomenon that in the presence of metal ions, these polyphenolic compounds can undergo redox cycling, leading to the generation of ROS [48]. Dual activity of AE can potentially be leveraged for therapeutic purposes, such as targeting cancer cells by inducing oxidative stress. The prooxidant properties of the extract can cause DNA damage in cancer cells, which was detected in our results. Particularly, the 0.5 mg DW/mL concentration of AE extract induced DNA damage to HT-29 cells that can be result of its prooxidant properties. The genotoxicity of AE methanol extract was shown also by Pukalskienė et al. [49]. Under certain conditions, antioxidants can have some additive effects during the oxidative damage. This can occur when antioxidants influence on signaling pathways stimulating prooxidant cellular mechanisms and leading to increased oxidative stress. A notable example of this phenomenon is the action of vitamin C, which, despite its widespread recognition as a potent antioxidant, can under certain circumstances act as a prooxidant, particularly in the presence of transition metals, leading to increased oxidative damage caused by hydrogen peroxide [50–52]. The possible genotoxicity in normal cells remains an important question that must be addressed in future toxicological assessments.

The phenomenon where antioxidants stimulate pro-oxidative cascades involves several cellular mechanisms and targets. Polyphenols can exert pro-oxidant effects through mechanisms such as redox cycling with transition metal ions (e.g., Fe³⁺ and Cu²⁺), which can catalyze the Fenton reaction to produce highly reactive hydroxyl radicals (•OH) that damage DNA, proteins, and lipids [53]. Furthermore, depending on their concentration, these compounds can undergo autoxidation upon reacting with oxygen, leading to the generation of reactive oxygen species (ROS) like superoxide anions (O₂•⁻) [54]. At high concentrations, antioxidants may overwhelm cellular defense systems, for instance by inhibiting the activity of protective enzymes like catalase or superoxide dismutase (SOD), leading to an accumulation of ROS [55]. This provides a strong theoretical basis for the genotoxicity observed in cancer cells treated with the AE extract, as this induced oxidative stress can directly lead to DNA damage [56].

The LC-Q-Orbitrap HRMS analysis revealed a presence various bioactive compounds discussed in Table 3. This comprehensive profiling not only confirms the complex chemical composition of *A. eupatoria* but also supports its traditional use in herbal medicine, providing a scientific basis for its application in modern therapeutics. The presence of bioactive compounds indicates potential health benefits, ranging from anti-inflammatory and antimicrobial effects to antioxidant and anticancer properties. The phytochemical characterization of AE extract growing in different regions of the world also done by several authors [5, 6, 11, 14, 57–60]. The presence of a wide variety of biologically active substances in AE extracts has already been demonstrated [6]. Despite numerous studies, none have annotated a higher number of phenolic compounds in AE extract than the presented study. Therefore, our results enhance the AE constituent database and can be important for a more comprehensive understanding of this herb’s bioactivities, potential mechanisms of action, and the roles and interactions of its various compounds.

**Table 3:** Major constituents identified in the aerial part ethanolic extract of *A. eupatoria* L. based on UV signal intensity and their potential biological activities according to the literature

Name	Class of compounds	Antioxidant activity*	Biological activities
Procyanidin dimer	Flavan-3-ols	+	Antitumor, antioxidant, antiviral, and anti-inflammatory [61]
Procyanidin trimer		+	
Procyanidin tetramer		+	
Procyanidin pentamer		+	
Catechin		+	Antioxidant, anticancer [25, 62]
Epicatechin		+	Antioxidant, anticancer [25, 62]
Epi/Catechin glucoside		+	N/A
3-O-Caffeoylquinic acid	Hydroxycinnamic acids	+	Antioxidant, anti-inflammatory, antibacterial, hepatoprotective, antidiabetic, anticancer, anticholinesterase and renoprotective activities [63–65]
p-Coumaroylquinic acid		+	Antioxidant, anticancer [66, 67]
Tricoumaroyl spermidine		–	Hepatoprotective [68]
Roseoside	Sesquiterpenoids	+	Anti-hypertensive, antioxidant, anti-inflammatory, and anti-vascular [69]
Taxifolin glucoside	Dihydroflavonols	+	Antidiabetic [70]
Pinoresinol	Lignan	–	Antitumor, chemopreventive, antifungal, anti-inflammatory, hypoglycaemic, chemopreventive [71]
Esculin	Coumarins	+	Anti-inflammatory, antioxidant, anti-diabetic, anticancer, antibiosis, antiviral, neuroprotive [72]
Quercetin derivatives (Quercetin-xylosyl-rutinoside, Quercetin-rutinoside, Quercetin-hexoside, Quercetin-malonyl-hexoside, Quercetin-rhamnoside)	Flavonols	+	Anticancer, antioxidant, and anti-inflammatory [73–76]
Agrimoniin	Ellagitannins	+	Anti-inflammatory, anticancer, immunomodulatory, antidiabetic, antibacterial, tyrosinase inhibiting, [10]
Kaempferol-rutinoside	Flavones	–	Anticancer, antioxidant, hepatorotective [77–79]
Luteolin-apiosyl-malonyl-glucoside		–	
Kaempferol-glucoside		–	Anticancer, antioxidant, hepatorotective [77–79]
Kaempferol-malonyl-hexoside		–	N/A
Apigenin-glucuronide		–	Anti-inflammatory, anti-complement, aldose reductase inhibitory [80]
Kaempferol-acetyl-glucoside		–	N/A
Tiliroside isomer		–	Anti-inflammatory, antioxidant, anticarcinogenic, and hepatoprotective activities [81]
Homoveratric acid	Phenylacetic acids	–	N/A
Trihydroxy-octadecenoic acid isomer	Fatty acids	–	N/A
Traumatic acid isomer		–	Antioxidant, anticancer [82, 83]
Phylloflavan	Cinchonains	–	Antiparasitic, antiviral [84]
Olivetolic acid	Hydroxybenzoic acids	–	Antibacterial [85]
Phloraspine	Phloroglucinols	–	N/A
Desaspidin/Flavaspidic acid		–	Antibacterial [86]
Aspidin		–	Antifungal, antibacterial, prooxidant, anticancer [87–89]
Agrimophol (6-[(3-butanoyl-2,6-dihydroxy-4-methoxy-5-methylphenyl)methyl]−3,5-dihydroxy-4,6-dimethyl-2-(2-methylbutanoyl)cyclohexa-2,4-dien-1-one)		–	Active ingredient of agrimony with anthelmintic, anticancer, Schistosomicidal, and mycobactericidal [90, 91]

* Antioxidant profiling was done by HPLC coupled post-column derivatization with ABTS reagent. “+” – presence of antioxidant activity, “-” – absence of antioxidant activity, “N/A” – not applicable

Molecular docking studies have provided insights into the binding affinities of AE major compounds with PI3K pathway. The data presented in this study reveal complex interrelationships between binding affinity and adherence to Lipinski’s rule of five, an established benchmark for evaluating drug-likeness. The compounds with the highest binding affinities to the Pi3K, such as quercetin xylosyl rutinoside, agrimoniin, and taxifolin glucoside, generally exhibited multiple violations of Lipinski’s rule. This trend suggests a potential trade-off between binding affinity and pharmacokinetic viability, an aspect that can significantly impact the progression of these compounds in drug development pipelines. These high-affinity compounds often have large molecular weights and a high number of hydrogen bond acceptors and donors. Such molecular characteristics are indicative of the ability to form multiple interactions within the protein binding pocket, which although beneficial for affinity, potentially hampers the bioavailability, permeability, and metabolic stability of these molecules. Indeed, the presence of multiple hydrogen bond donors and acceptors can enhance water solubility but may also increase the likelihood of being a substrate for efflux transporters or being metabolically unstable, thus reducing their overall effectiveness as drug candidates [92].

Conversely, the compounds that adhere strictly to Lipinski’s rule of five generally showed lower binding affinities than the reference compound. This observation aligns with the notion that while such compounds are more likely to possess favorable drug-like properties, they may lack sufficient binding interactions necessary for effective target modulation. This dichotomy highlights the inherent challenges in drug discovery, where optimal binding affinity must be balanced against pharmacokinetic properties to ensure both efficacy and safety.

However, tricoumaroyl spermidine stands out as a noteworthy exception. This compound not only demonstrated a better binding affinity than the reference inhibitor but also maintained minimal deviation from the established drug-likeness criteria, violating only one rule concerning molecular weight, which marginally exceeds the threshold set by Lipinski’s rule. Interestingly, its molecular weight is close to 500, which may not significantly impact its pharmacokinetic properties, suggesting a manageable alteration for optimization. The fact that tricoumaroyl spermidine aligns closely with Lipinski’s parameters while still exhibiting strong binding affinity makes it a promising candidate for further investigation.

The unique profile of tricoumaroyl spermidine suggests that it could maintain adequate bioavailability and metabolic stability while effectively engaging with the target protein, an ideal scenario in drug design.

Computational analysis of tricoumaroyl spermidine PI3K interaction, revealed compelling visual evidence of the potential for this compound to act as a potential PI3K inhibitor. The optimal docking pose, characterized by the close and extensive interactions with the enzyme’s active site, supports the computational predictions and the observed binding affinity data. This fit is not merely superficial but is supported by a range of interactions that stabilize the molecule within the binding pocket, which is essential for effective inhibition. Despite its slightly larger molecular weight, which slightly exceeds the typical cutoff in Lipinski’s rule of five, the compound’s configuration enables it to maintain a high level of interaction without significant steric hindrance or unfavorable dynamics that might impede its binding. This balance between size, functionality, and fit within the binding site is crucial and suggests that tricoumaroyl spermidine might retain good pharmacokinetic properties despite its deviation from one of Lipinski’s rules.

Furthermore, the computational predictions underscore the multifaceted interaction profile of tricoumaroyl spermidine with the PI3K protein, which is critical for its enhanced binding affinity. The hydrogen bonds, especially those with key residues like ASP950 and GLU880, are indicative of strong and specific interactions that are likely to contribute to the efficacy of tricoumaroyl spermidine as a kinase inhibitor. The presence of multiple interaction types, including pi interactions and hydrogen bonds, suggests that tricoumaroyl spermidine is able to form a complex yet stable interaction network within the binding pocket of PI3K. The additional interactions, particularly pi-alkyl and pi-anion, may compensate for any potential instability caused by the unfavorable donor-donor interactions, highlighting a balanced interaction profile conducive to high binding efficiency. Structural adjustments could potentially reduce unfavorable interactions and enhance favorable ones, optimizing both binding affinity and drug-like characteristics without significantly altering the compound’s core structure. This approach could lead to the development of a more potential and selective PI3K inhibitor based on the tricoumaroyl spermidine scaffold, warranting further investigation into its therapeutic potential.

Based on literature tricoumaroyl spermidine has been found in several foods, such as winter squash, red rice, common pea, and eggplant (https://hmdb.ca/metabolites/HMDB0304518), in flowers of Tieguanyin tea [93], Potentilla species [94], as well as in Castanopsis honey [95]. Remarkable hepatoprotective activity of tricoumaroyl spermidine from a rose was shown in HepG2 cells[68]. There is no other literature evidence about other bioactivities of this compound.

### Limitations of the study

This study has several limitations that should be acknowledged. First, the plant material was sourced from a single geographical region in Armenia, which may not account for the potential chemical variability of *A. eupatoria* L. from other locations. Second, while our findings on cytotoxicity and PI3K inhibition are promising, the study lacks comprehensive in vivo data, and further investigations into the toxicity profiles and pharmacokinetic properties of both the crude extract and the isolated tricoumaroyl spermidine are necessary. Future investigations should also include phospho-specific antibodies (e.g., p-Akt Ser473, p-mTOR Ser2448) to fully characterize the activation state of the PI3K/Akt/mTOR pathway under treatment conditions. While our study provides preliminary evidence of apoptosis via Hoechst staining and total caspase-3 upregulation, further confirmation using cleaved caspase-3 detection and Annexin V/PI flow cytometry is necessary. These will be priorities for our planned future studies. Additionally, the observed pro-oxidant effects and modest genotoxicity warrant further investigation in normal, non-cancerous cells to fully assess the therapeutic window. Finally, although tricoumaroyl spermidine was identified as a strong potential inhibitor, the overall biological activity of the extract is likely due to the synergistic effects of multiple constituents, and other identified compounds also merit further experimental validation.

## Conclusions

In conclusion, *Agrimonia eupatoria* L. ethanolic extract demonstrates a multi-dimensional anti-cancer effect, stemming from its rich phenolic composition which confers cytotoxic activity and induces oxidative stress in cancer cells. The primary mechanism we validated is the potent inhibition of the PI3K/Akt/mTOR signaling pathway in A549 cells. Our experimental data confirms that the extract significantly reduces the expression of PI3K, Akt, and mTOR, as well as downstream effectors linked to hypoxia (HIF-1α), inflammation (COX-2), and metastatic potential (MMP-2). The bioactive molecule tricoumaroyl spermidine, identified through computational analysis, is a promising candidate likely involved for these effects. Despite its moderate direct cytotoxicity, AE extract acts as a pathway-modulating agent, enhancing apoptosis and suggesting its potential as an adjunct to existing therapies. These findings warrant further in vivo validation and the isolation of its key compounds to fully develop integrative phytochemical strategies for cancer treatment.

## Supplementary Information

Supplementary Material 1.

## Data Availability

The datasets and materials used and/or analyzed during the current study are available from the corresponding author upon reasonable request. The raw LC-HRMS/MS spectra are available from the corresponding author upon reasonable request.

## Abbreviations

AE: Agrimonia eupatoria
PI3K: Phosphoinositide 3-kinase
LC-Q-Orbitrap HRMS: Liquid Chromatography-Quadrupole-Orbitrap High-Resolution Mass Spectrometry
ABTS: 2,2’-Azino-bis(3-ethylbenzothiazoline-6-sulfonic acid)
DPPH: 2,2-Diphenyl-1-picrylhydrazyl
MTT: 3-(4,5-Dimethylthiazol-2-yl)-2,5-Diphenyltetrazolium Bromide
ELISA: Enzyme-Linked Immunosorbent Assay
DNA: Deoxyribonucleic Acid
DW: Dry Weight
HBA: Hydroxybenzoic Acids
HCA: Hydroxycinnamic Acids
