# Supplementary material for: Inhibition of the PI3K signaling pathway in cancer cells by Agrimonia eupatoria L. ethanolic extract: identification of tricoumaroyl spermidine as a potential PI3K inhibitor
Source: BMC Complement Med Ther. 2026 Jan 6;26:41. doi: 10.1186/s12906-025-05231-z (PMC12870893; doi:10.1186/s12906-025-05231-z)

**Uncropped Gels and Blots**

The uncropped images of the blots presented in **Figure 6B** are provided in this supplementary file. Each nitrocellulose membrane was developed for two target proteins by physically dividing the membrane into two parts based on the expected molecular weights of the respective targets.


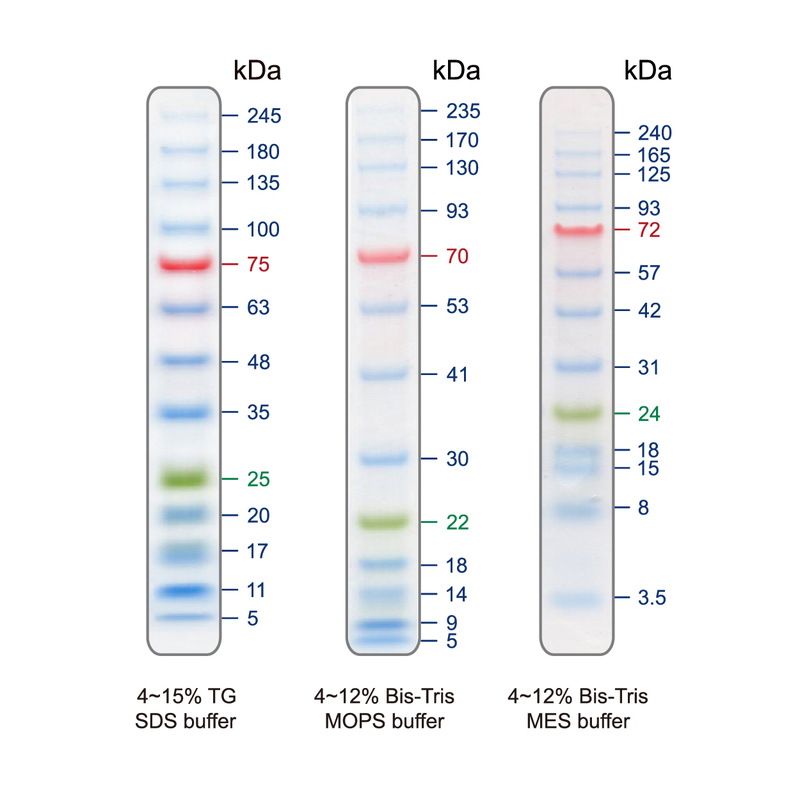

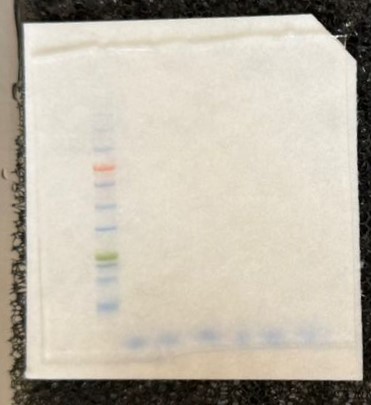


**Gel 1 – mTOR and Akt**


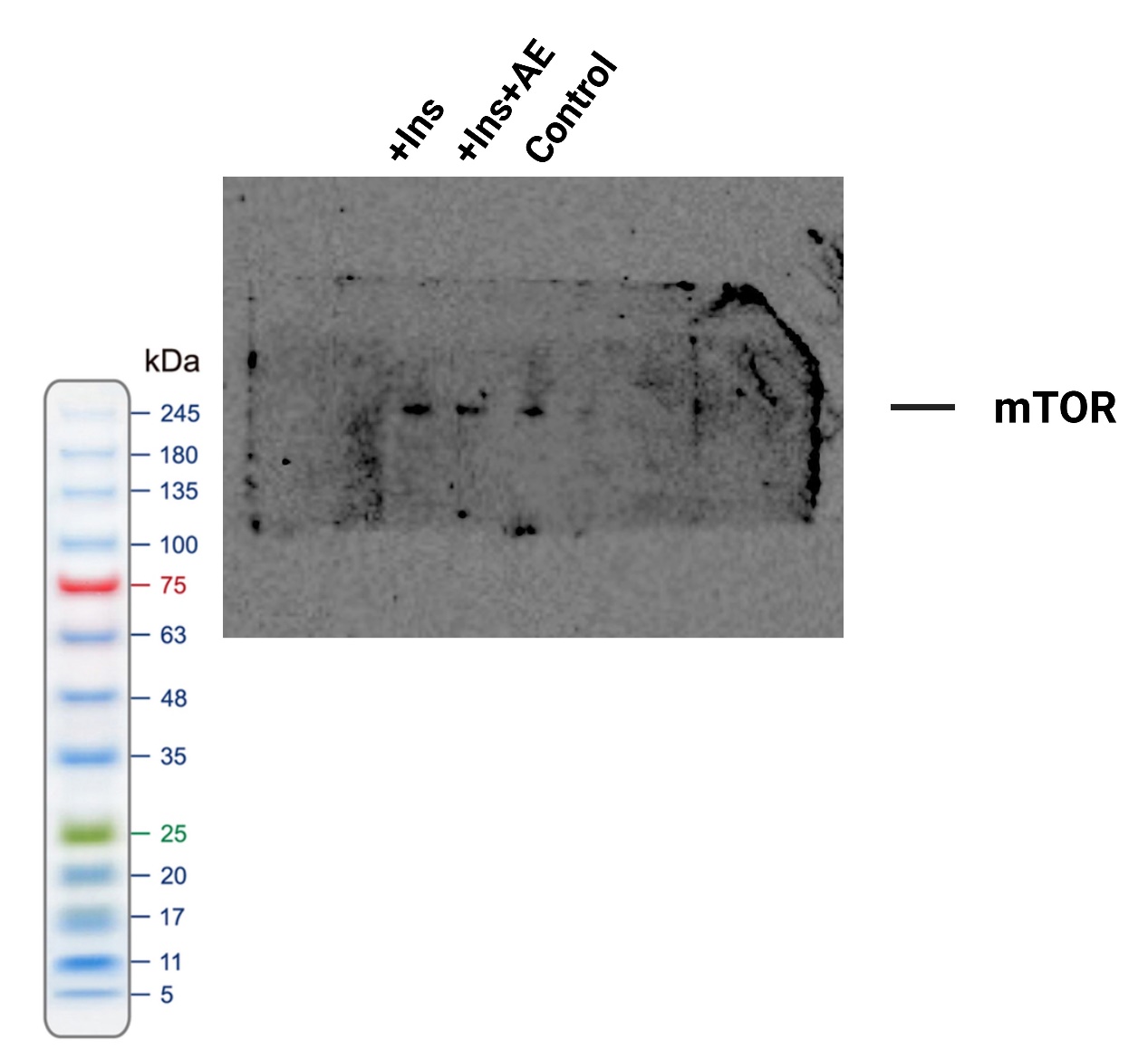


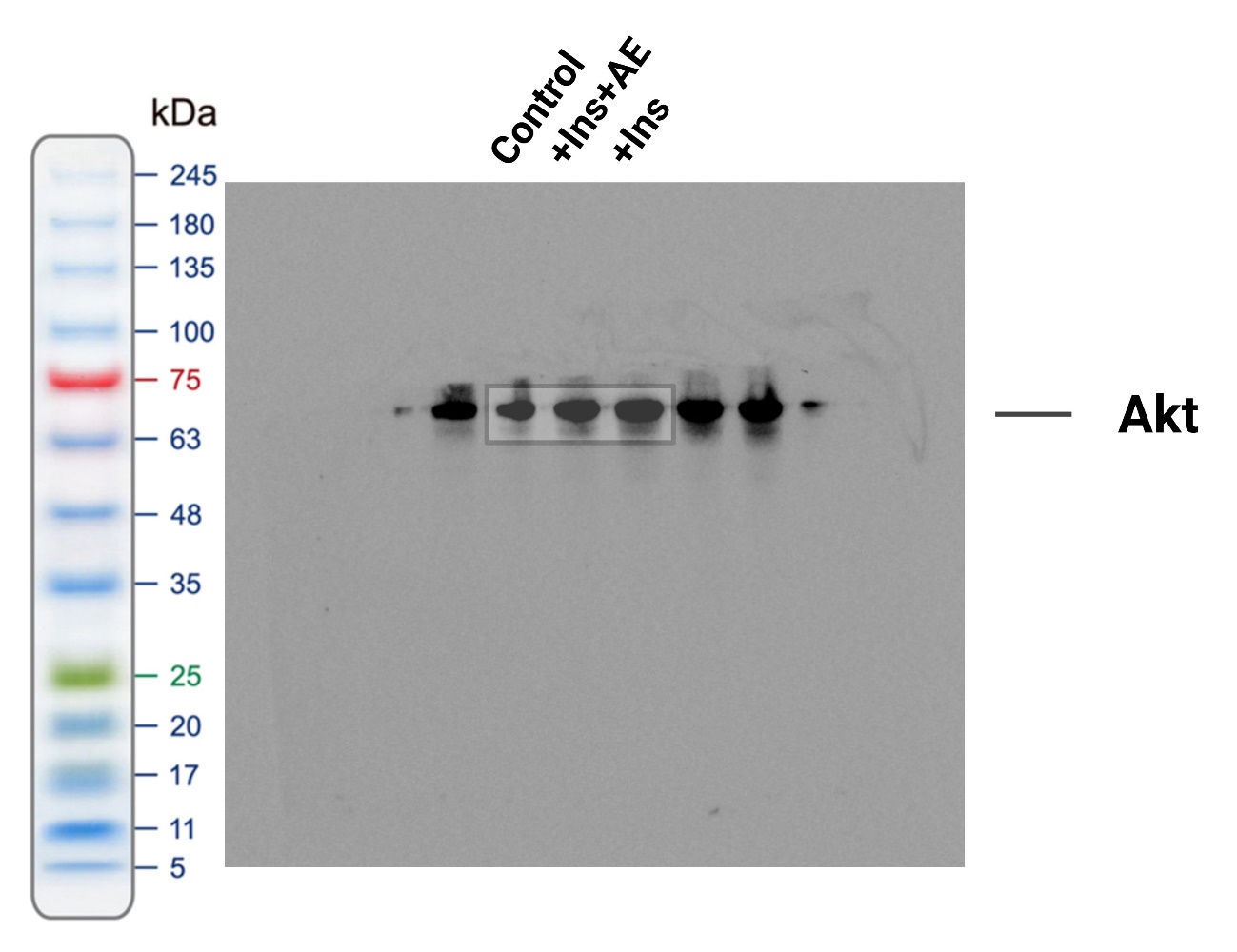


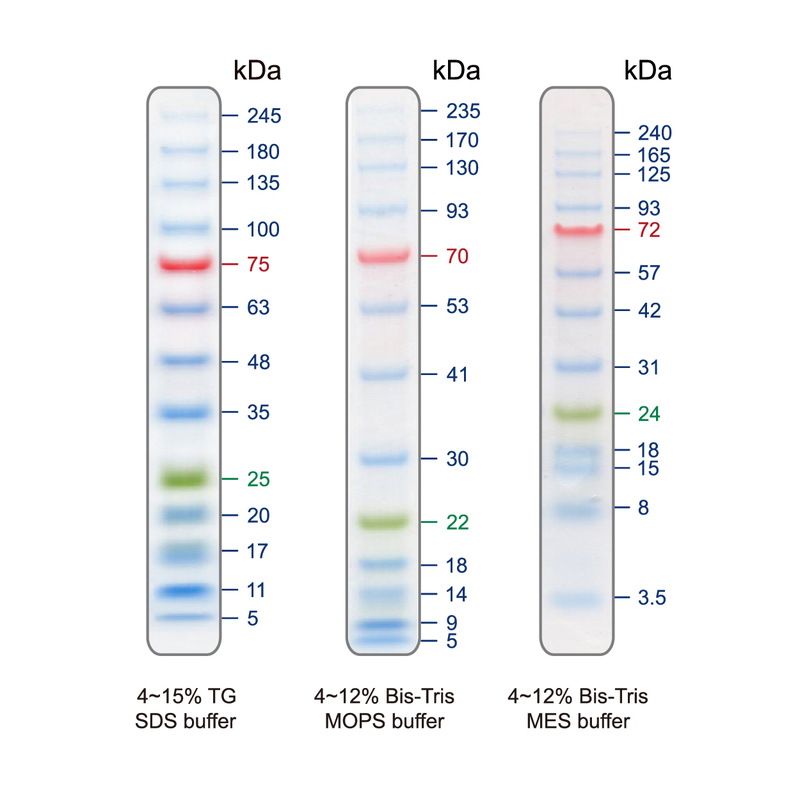

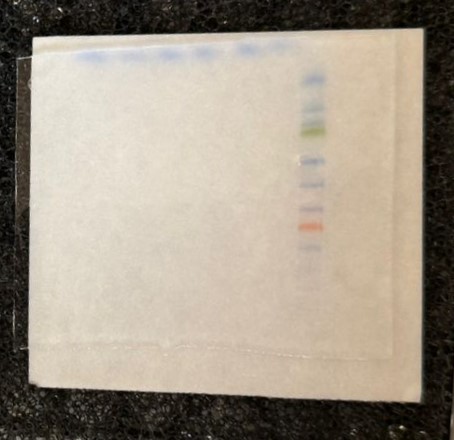


**Gel 2 -PI3K and actin**


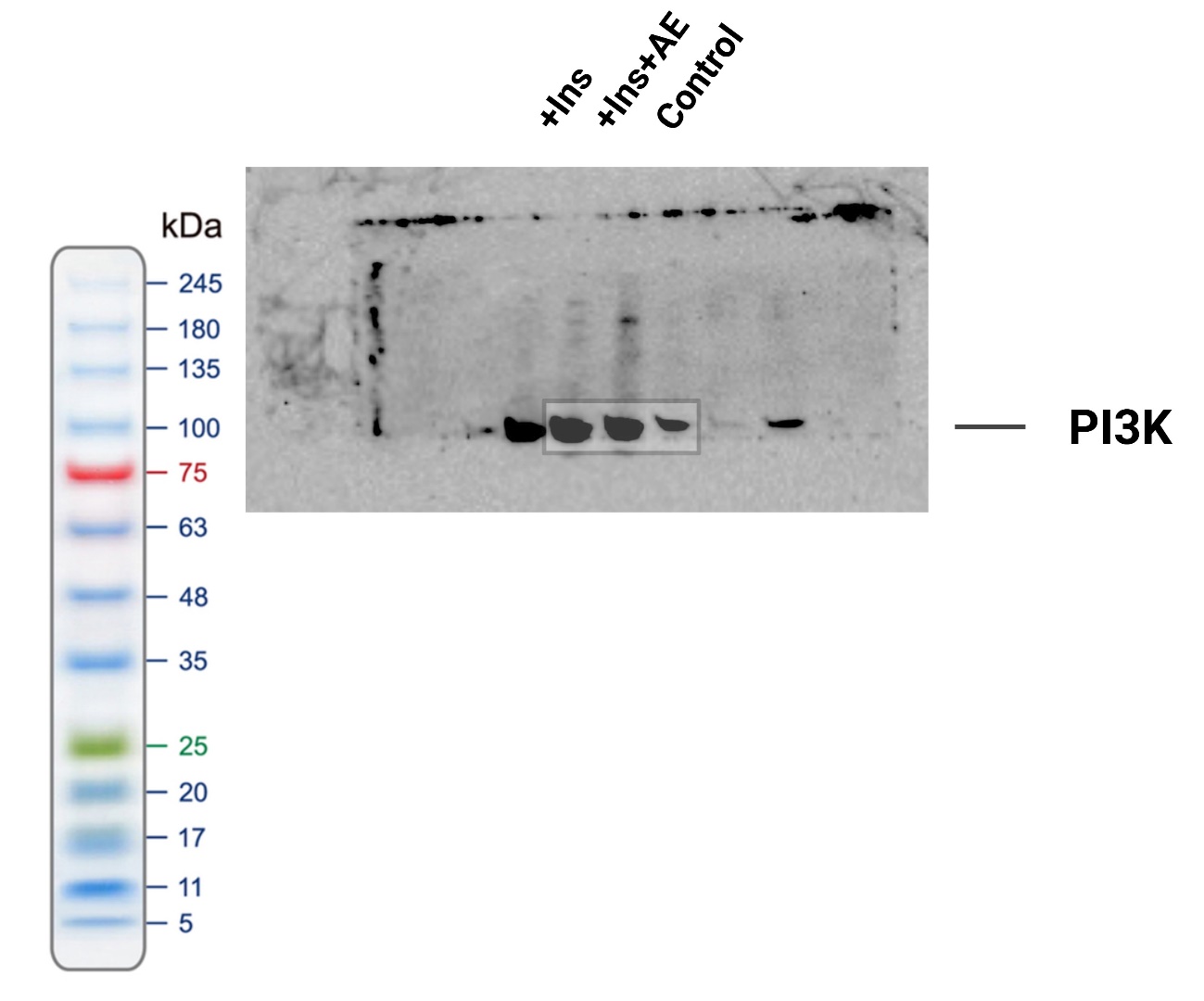


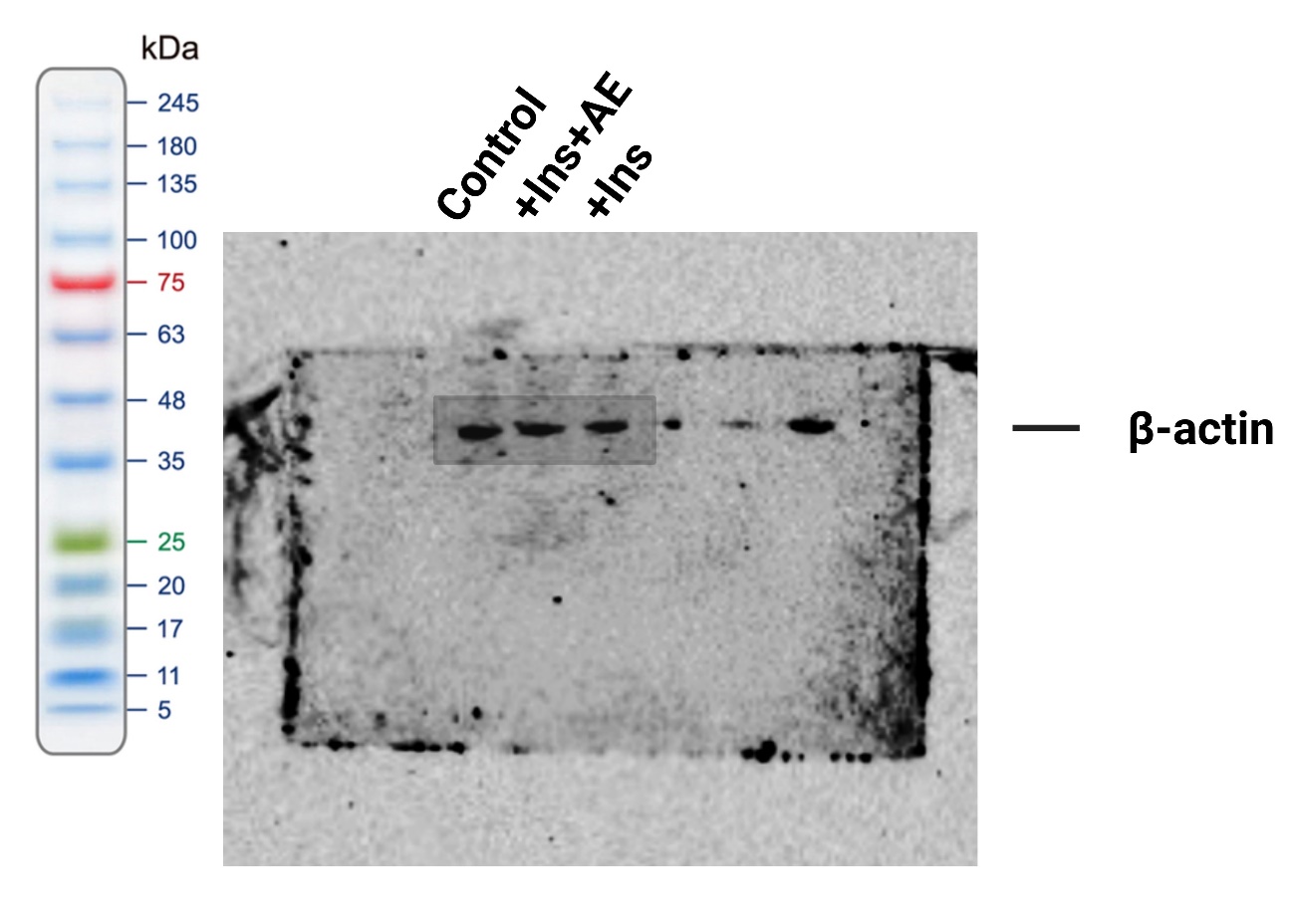


**2nd and 3rd repetitions**


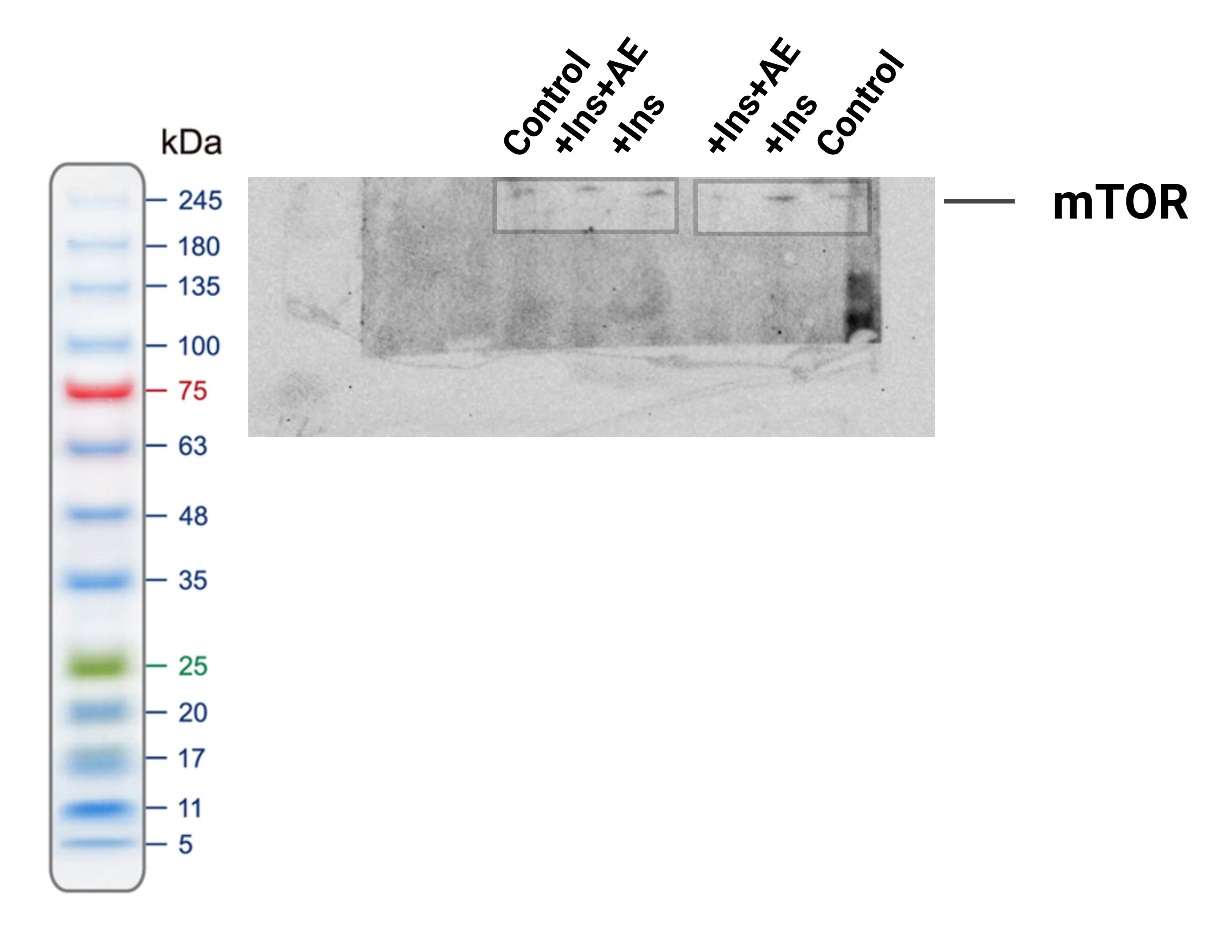

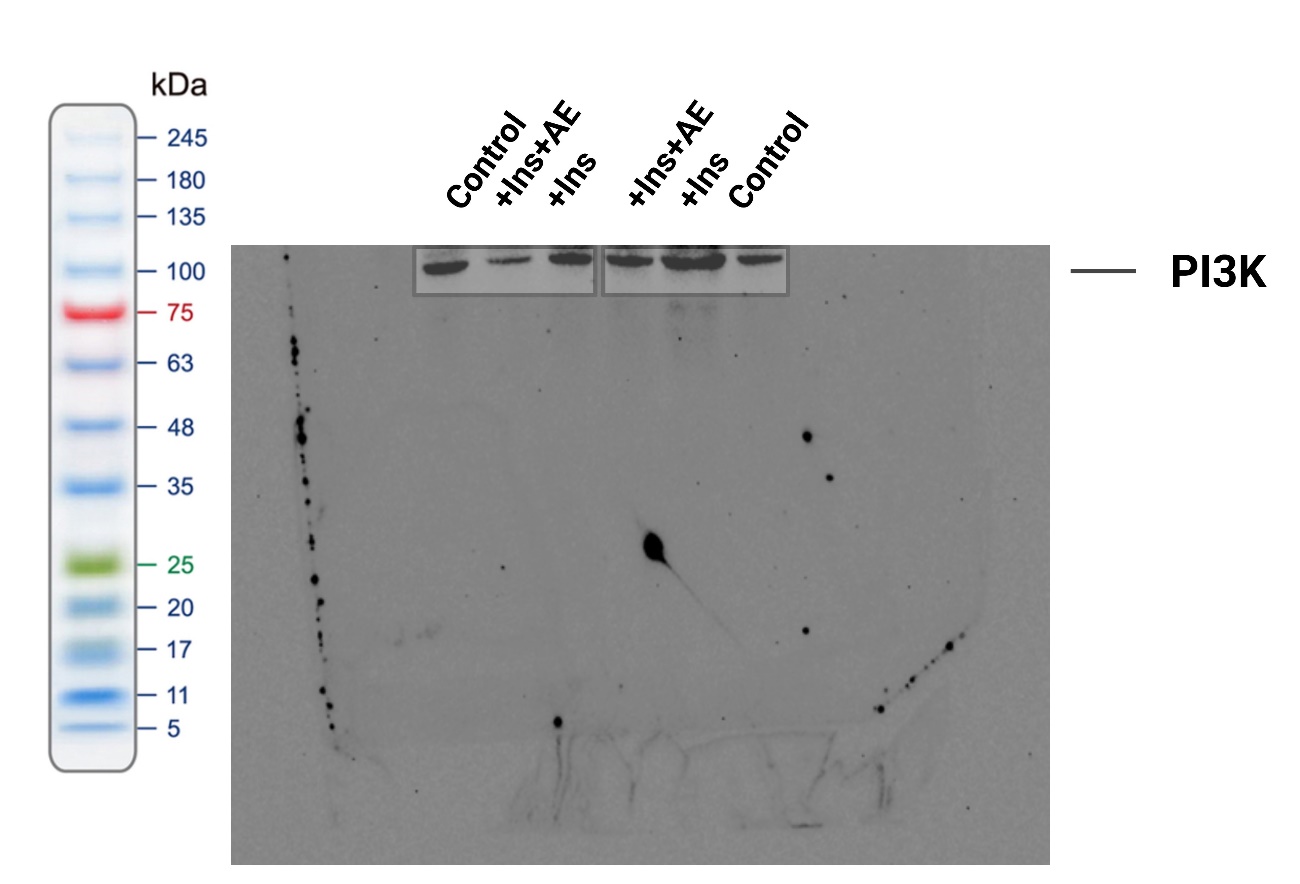


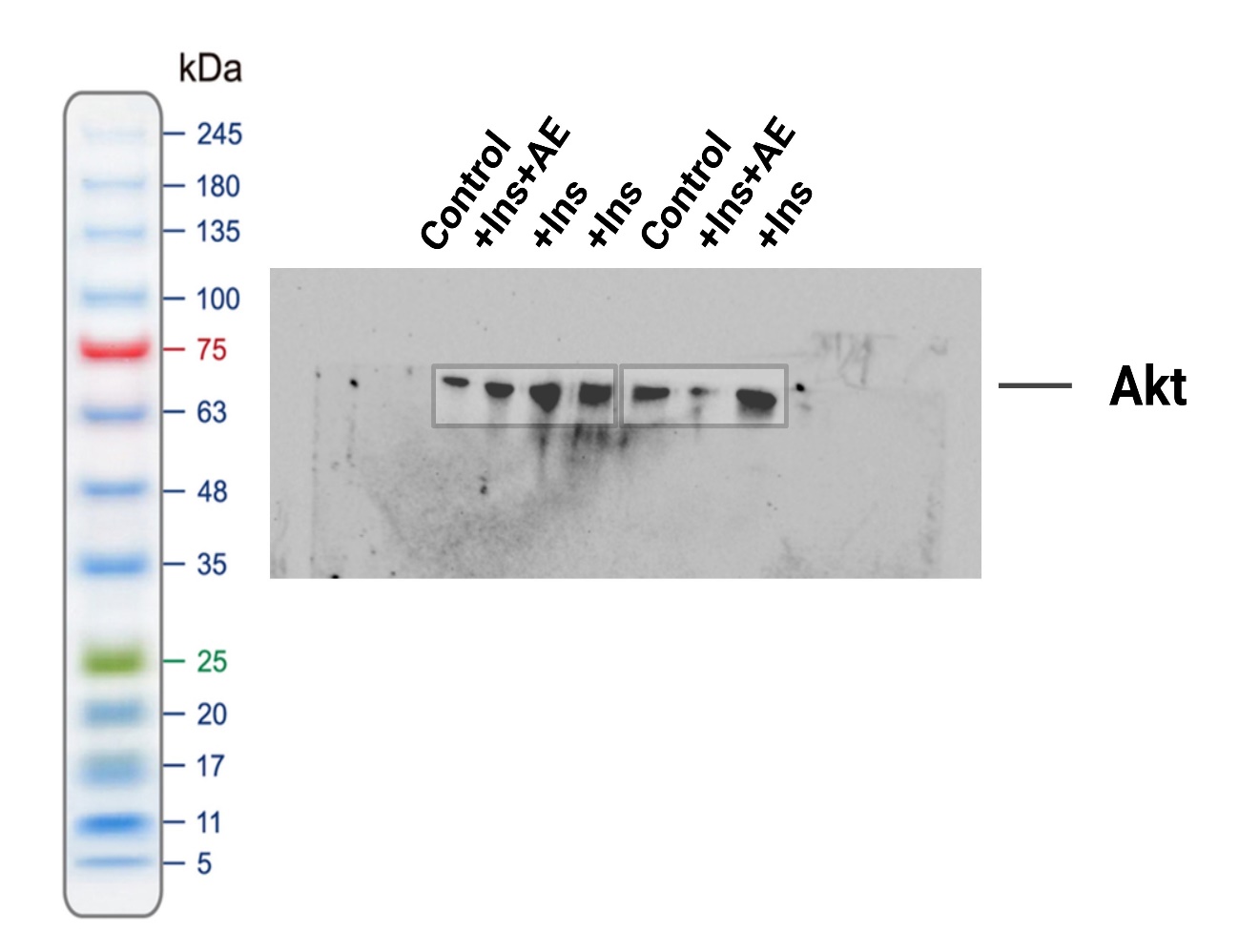


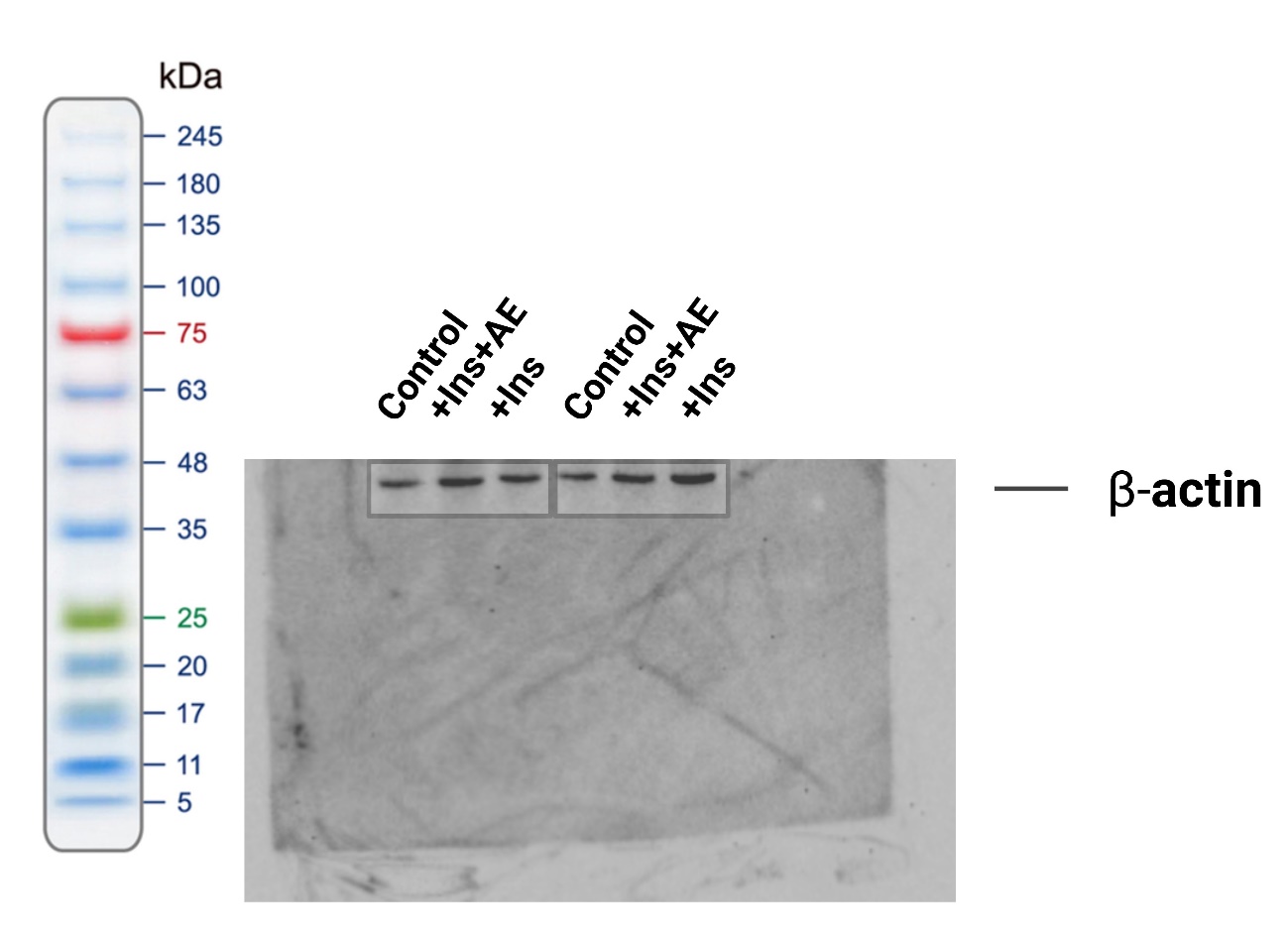

Supplement: Supplementary file 1 — Supplementary Material 1. [file 12906_2025_5231_MOESM1_ESM.docx]
